# Robotic wireless capsule endoscopy: recent advances and upcoming technologies

**DOI:** 10.1038/s41467-024-49019-0

**Published:** 2024-05-30

**Authors:** Qing Cao, Runyi Deng, Yue Pan, Ruijie Liu, Yicheng Chen, Guofang Gong, Jun Zou, Huayong Yang, Dong Han

**Affiliations:** 1 State Key Laboratory of Fluid Power and Mechatronic Systems, Zhejiang University, Hangzhou, 310027 China; 2 School of Mechanical Engineering, Zhejiang University, Hangzhou, 310027 China; 3 Sir Run-Run Shaw Hospital, College of Medicine, Zhejiang University, Hangzhou, 310016 China

**Keywords:** Gastrointestinal system, Biomedical engineering

## Abstract

Wireless capsule endoscopy (WCE) offers a non-invasive evaluation of the digestive system, eliminating the need for sedation and the risks associated with conventional endoscopic procedures. Its significance lies in diagnosing gastrointestinal tissue irregularities, especially in the small intestine. However, existing commercial WCE devices face limitations, such as the absence of autonomous lesion detection and treatment capabilities. Recent advancements in micro-electromechanical fabrication and computational methods have led to extensive research in sophisticated technology integration into commercial capsule endoscopes, intending to supersede wired endoscopes. This Review discusses the future requirements for intelligent capsule robots, providing a comparative evaluation of various methods’ merits and disadvantages, and highlighting recent developments in six technologies relevant to WCE. These include near-field wireless power transmission, magnetic field active drive, ultra-wideband/intrabody communication, hybrid localization, AI-based autonomous lesion detection, and magnetic-controlled diagnosis and treatment. Moreover, we explore the feasibility for future “capsule surgeons”.

## Abstract (web summary)

future requirements for intelligent wireless capsule endoscopy, providing a comparative evaluation of various methods’ merits and disadvantages, and highlighting recent developments in six technologies.

## Introduction

Medical endoscopes play a pivotal role in diagnosing gastrointestinal abnormalities, including gastric polyps, gastrointestinal bleeding, and Crohn’s disease^1^. Traditional endoscopic examinations require doctors to insert a long cable into the patient’s body cavity for imaging crucial areas and providing diagnostic and therapeutic solutions. Nevertheless, such wired endoscopes, due to their voluminous contact area, may cause discomfort and pain to patients. They also pose potential complications like infection, perforation, and tearing, and have a notable inability to reach extensive areas of the small intestine^2,3^. In contrast, wireless capsule endoscopy (WCE) is globally recognized for its patient-centered approach and non-invasive nature, offering an effective alternative that effectively avoids the aforementioned issues^4^.

WCE persists as a rapidly advancing and highly impactful field of research, emerging as one of the eight key research topics in medical robotics from 2010 to 2020^5^. The recent study^6^ has confirmed the critical role of WCE devices in navigating acute gastrointestinal bleeding, even in resource-limited environments like the COVID-19 pandemic. Unlike traditional endoscopy, WCE eliminates the need for anesthesia and replicates the ingestion process of standard capsule medication. It moves passively or actively through the gastrointestinal tract, capturing images at a controlled pace. The video signals are wirelessly transmitted to a receiver affixed to the patient, allowing medical professionals to identify potential anomalies for diagnosis.

**Fig. 1:**
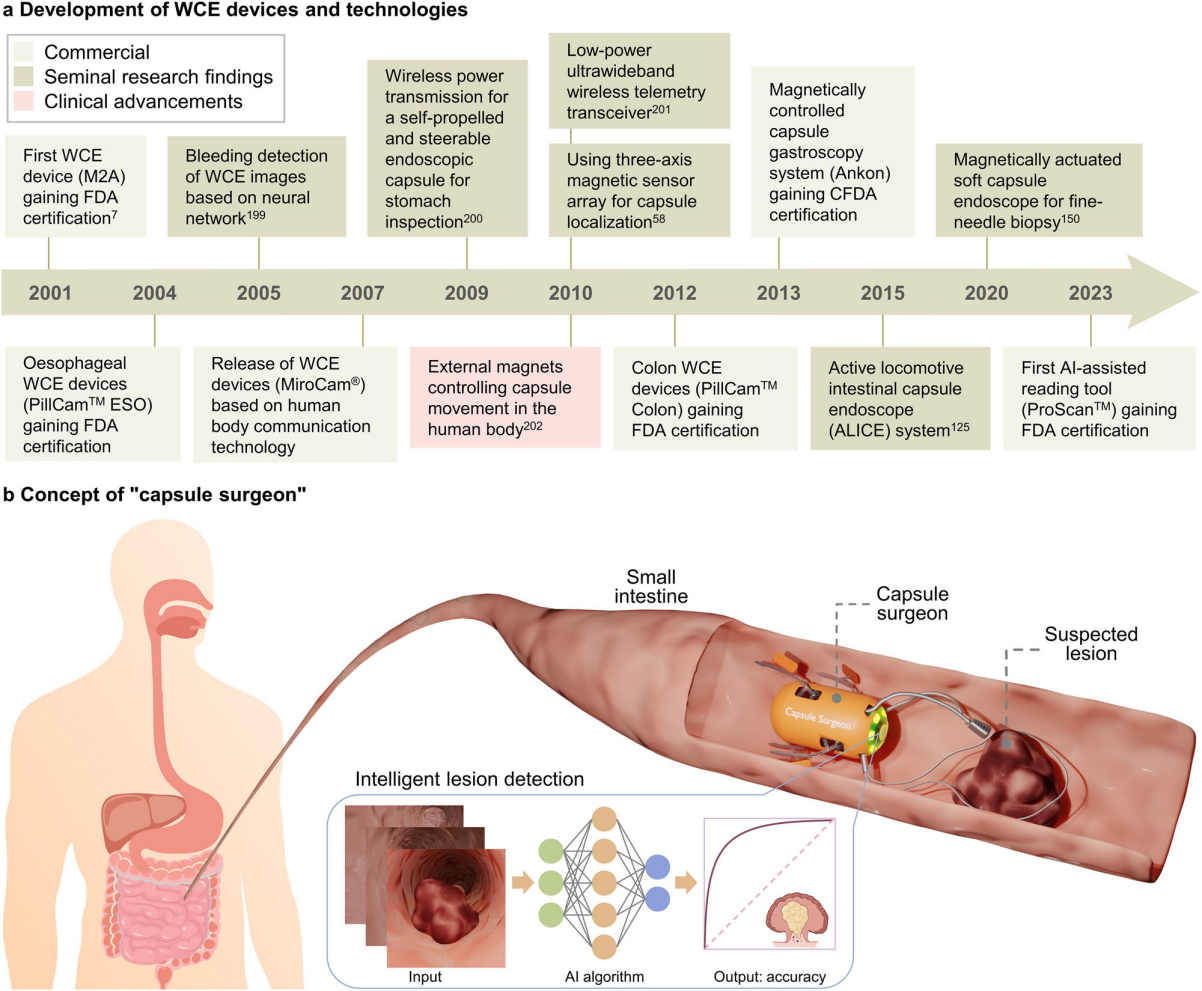
Timeline of major milestones in the development of WCE robots and their future conceptual design. **a** Development of WCE devices and technologies^7,58,125,150,199–202^. The timeline illustrates the commercial progress of WCE devices, seminal research findings, and clinical advancements from 2001 to 2023. WCE wireless capsule endoscopy, FDA US Food and Drug Administration, CFDA China Food and Drug Administration. **b** Concept of “capsule surgeon”. Utilizing intelligent detection algorithms, the capsule robot undertakes inspections for potential lesions. Upon identifying abnormal tissue, the capsule can secure its position and kickstart medicinal procedures with its functional components. These elements are manifold and might involve snares, high-frequency electrotomes, or hemostatic clips. AI artificial intelligence. Figure 1, created with BioRender.com, released under a Creative Commons Attribution-NonCommercial-NoDerivs 4.0 International license. Partial image elements designed by brgfx - Freepik.com.

Standard WCE devices are typically 11$\times$26 mm in size and incorporate components such as a lens, image sensor, LED, button battery, and antennas^2^. Since the first commercial capsule endoscope, M2A (renamed as PillCam^TM^ SB), gained medical certification in 2001, WCE technology has evolved over two decades^7^ (Fig. 1a). Various models of commercial capsule endoscopes are currently accessible in the market (Supplementary Table 1). Improvement has not been confined to enhancing image quality and extending battery life. Some capsules are designed to maximize examination completion rates through active movement, and also to conduct intelligent lesion detection^8,9^.

Despite being widely used in clinical practice for small intestine examination, commercial capsules have a few limitations. For instance, they are not suitable for surgical interventions during diagnostic procedures and have limited endurance. Therefore, they cannot completely replace wired flexible endoscopy. Recent advancements in emerging technologies, such as micro-electro-mechanical systems (MEMS) and artificial intelligence (AI), have prompted new criteria for the development of the next generation of intelligent capsule endoscopy robots:

1. High endurance: ensures capsule endoscope capability for real-time imaging, data transmission, and observation from ingestion through the full passageway of the digestive system.
2. Active motion navigation: alleviates manual operation by facilitating the remote control of capsule position and direction within the cavity, promising a broad field of view and minimizing chances for missed inspection.
3. High-speed bi-directional communication: enables WCE devices to transmit images or sensor data to the receiver, as well as receive control commands for specific functions.
4. High-precision positioning: allows medical practitioners to accurately locate lesions in real-time, which aids subsequent diagnosis and treatment.
5. Intelligent lesion detection: capsule endoscopes come with built-in functions to automatically identify lesions, effectively reducing the time doctors spend on inspection.
6. Integrated diagnostic and therapeutic function: WCE robots can visually identify and perform minimally invasive surgeries on lesions without any skin damage.

Aligning with the concept of “swallowing the surgeon” proposed by Richard Feynman in his 1959 lecture “There’s plenty of room at the bottom“^10^, a new generation of intelligent capsule endoscopy robots that fulfill the specified criteria can autonomously execute surgeries within the human body, eliminating the need for direct intervention by the doctor (Fig. 1b).

This review provides a comprehensive comparison of technologies associated with the “capsule surgeon” concept. It explores the state-of-the-art intelligent technologies utilized by WCE robots and suggests potential future directions for capsule endoscopy research.

## Technical comparison of WCE

**Fig. 2:**
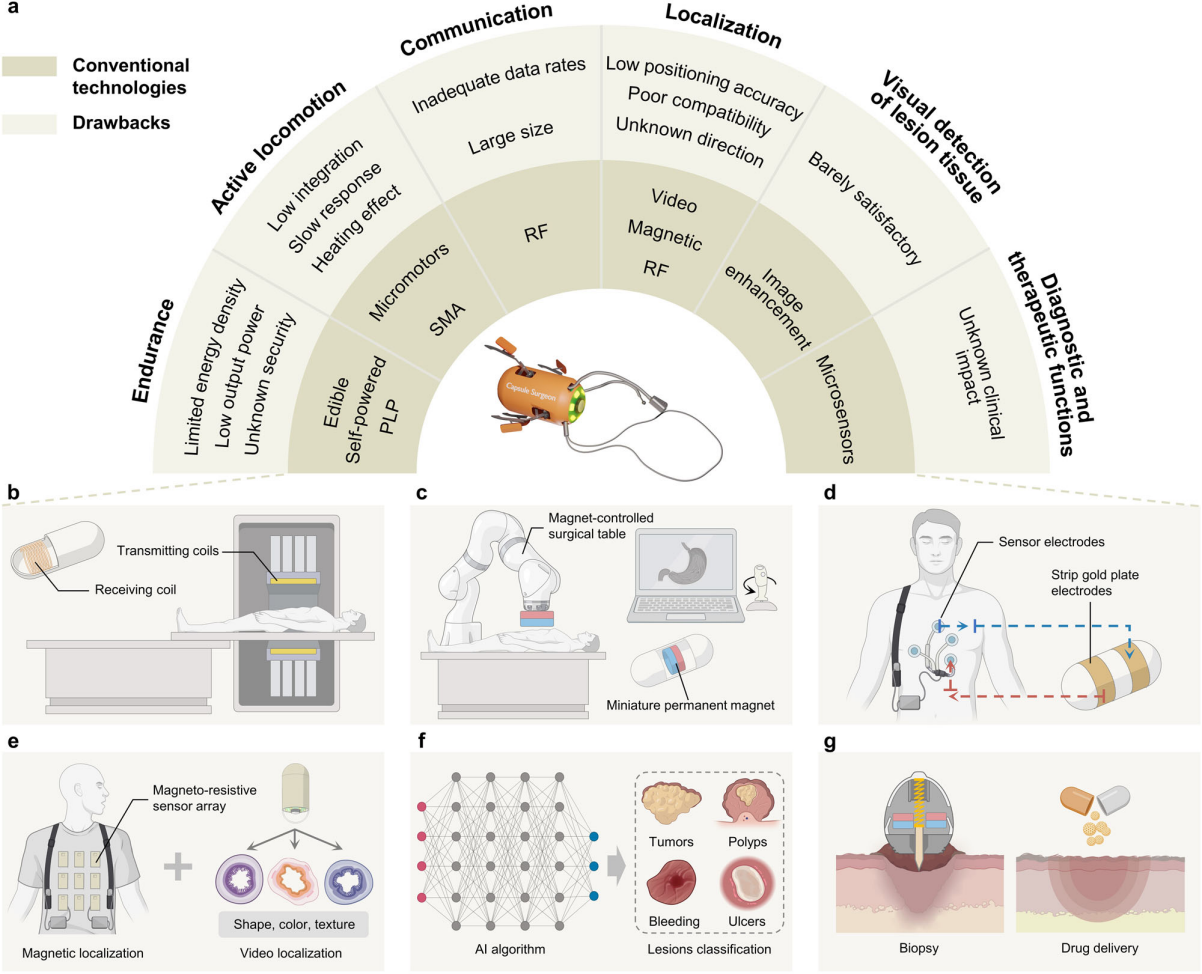
Traditional technology defects and advanced intelligent technologies of WCE. **a** Drawbacks of conventional methods. PLP packaged lithium-ion polymers, SMA shape memory alloy, RF radio frequency. **b**–**g** Working principles of advanced intelligent technologies. **b** Near-field wireless power transmission. The patient lies on an operating table with a transmitting coil and a capsule containing the receiving coil in the body. **c** Magnetic field active drive. The physician manipulates a robotic arm endowed with an external magnet, through a handle, dictating the motility of an internal capsule containing a magnet. **d** Intrabody communication. A wearable receiver containing multiple electrodes is looped with two strip gold electrodes on the capsule. **e** Magnet/video hybrid location. Magnetic positioning depends on an external magneto-resistive sensor array, and video positioning employs tissue features such as shape, color, and texture for identification. **f** AI algorithm achieves lesion classification. **g** Magnetic field-controlled capsules perform diagnostic and therapeutic functions, including biopsy or drug release at targeted locations. Figure 2, created with BioRender.com, released under a Creative Commons Attribution-NonCommercial-NoDerivs 4.0 International license.

We have conducted an extensive search within the Web of Science database for research literature published since 2000, highlighting six facets of WCE: endurance, active locomotion, communication, localization, visual detection of lesion tissue, and diagnostic and therapeutic functions. This section aims to draw a comparison between the setbacks of traditional technologies and identify the most suitable intelligent alternatives that fulfill the surgical requirements of a “capsule surgeon” (Fig. 2a), as per our perspective. An in-depth discussion of recent advancements in these described technologies is mentioned in the next section.

### Endurance

The longevity of the WCE system depends greatly on maintaining a high level of integration. Most capsules house two silver oxide button batteries. At a power rating of approximately 20 mW, they can meet the energy demand of the capsule’s internal circuit for 8–10 h under a voltage of 3V^11^. However, current commercial battery solutions leave room for improvement when it comes to supporting the expansion of capsule functions. When choosing a new power source, key factors such as battery capacity, power output, size, and safety must be taken into consideration.

WCE could benefit from integrating custom-shaped lithium-ion polymers, which could potentially extend battery life and augment power^12^. These polymers’ power densities are 2000 times greater than alternatives, providing high peak current, but safety considerations warrant further analysis due to frequent temperature runaway issues^13^.

A prospective solution could be a new form of self-powered battery that directly harnesses the digestive system’s energy, transforming gastric fluid into a virtually unlimited electrolyte supply to extend battery life^14,15^. Nonetheless, it also faces a major hurdle—addressing the issue of low output power.

Edible electronics, made from food-grade materials safe for human consumption, have gained significant interest due to their superior safety and non-invasive advantages. These devices use energy storage or harvesting techniques such as edible batteries, supercapacitors (SCs), and nanogenerators^16^, all of which are compatible with WCE. One recent study by Ilic et al.^17^ introduced an edible rechargeable battery, composed of common food ingredients and additives, leveraging redox cofactors to power biochemical devices. Despite being biodegradable and rechargeable, the edible battery’s application is limited by its low power density. Edible SCs store and release energy through charge separation at the electrode-electrolyte interface. They represent an advancement over traditional capacitors, offering high power output comparable to commercial button batteries and demonstrating excellent cycle life. However, SCs continue to encounter challenges related to low energy density^18,19^. Nanogenerators are energy collectors that convert minute forces or thermal energy (like vibrations, pressure, and heat) in the environment into electrical energy^20^. Similar to edible batteries, they cannot deliver high power output currently, only suitable to provide energy for monitoring miniature medical devices^21^.

In response to the complexities of battery development, researchers are investigating the potential of battery-free wireless power transmission (WPT) technology as a viable solution for WCE. The WPT system converts electromagnetic waves into energy, enabling a wireless power transfer from the transmitter to the receiver. There are two types of WPT technology, distinguishable by the coupling region between the transmitting and receiving antennas: near-field transmission (non-radiative) and far-field transmission (radiative)^22^. Of these, near-field transmission stands out for its high efficiency over short distances, making it a valuable tool in a host of implantable medical devices, including left ventricular assist devices, heart rate sensors, and deep brain stimulators^23^. Previous findings concerning the safety, efficiency, and comfort of near-field WPT provide promising insights into their potential usefulness in powering WCE systems.

Typically, near-field WPT integrates a small receiving coil (RC) within the capsule and coupling it with a large transmitting coil (TC) placed on the external operating table (Fig. 2b). Conceptual models such as the sliding clamper-style capsule robot and the tether-free inchworm-like capsule robot, proposed by Gao et al., incorporate a cylindrical RC into the capsule^24,25^. When utilizing near-field WPT, they demonstrated a remarkable active movement speed of 6.32 cm/min. According to literature^26^, the power transmission capacity of near-field WPT could peak at 500 mW. This suggests that it allows the effective use of the capsule’s internal space for other functional modules while providing a dependable power supply to meet the capsule’s power needs.

### Active locomotion

The exploration process employed by WCE is inherently passive and relies solely on the spontaneous peristalsis of the gastrointestinal tract for progression. This inherent limitation precludes a more extensive examination of specific sites within the gastrointestinal tract, contributing to an appreciable omission rate of up to 30%^27^. Additionally, the study^28^ further indicates that slower gastric transit times, under conditions of passive movement, could notably increase the risk of capsule retention in the stomach. Consequently, the design and implementation of an active motion mechanism to control the capsule’s movement is of paramount importance. Active locomotion of WCE hinges upon a reliable driving source. However, designing an efficient power transmission mechanism within the limited space available presents a significant challenge.

Initial studies have delved into the usage of shape memory alloy (SMA) to convert electrical energy into mechanical deformation, intending to develop a legged capsule robot that mimics crawling creatures^29–31^. The approach accounts for the necessity of compactness, high power density, and low driving voltage in WCE. Nonetheless, the driving mechanism of SMA inevitably results in environmental heating and delayed response.

Micromotors could serve as power sources and, in conjunction with jigs, enable stable forward movement by switching between linear and expansion anchoring modes^24,25,32^. Due to its incorporation of multiple intricate mechanical components, this method is typically only suitable for colonic environments with larger cavity sizes. Another “micro submarine” presents a suitable instance with a capsule size consistent with clinical applications. Utilizing the motor’s propeller to exert fluid pressure and generate a reactionary force, it can achieve multi-directional propulsion in the stomach^33^, or high-speed transit in the simulated intestine^34^. Nevertheless, integration and endurance currently restrict the ability of a single capsule to conduct comprehensive motion exploration within the digestive system, thus impeding the widespread adoption of this micro-motor technology.

Magnetic field control presents a highly promising avenue. Offering unique advantages such as contactless operation, durability, and reliability, it’s the ideal alternative for governing miniature robots within the human body. The breadth of its applications spans various medical procedures, including minimally invasive brain surgery, bronchoscopic lung examination, and magnetic guidewire vascular intervention^3^. Since Carpi et al. ^35^. Introduced the concept of maneuvering a capsule endoscope through magnetic interactions in 2006, hundreds of research papers on magnetically controlled capsule robots have emerged. A notable milestone was the development of the first clinical system pilot, which was based on the collaboration between Olympus and Siemens and utilized a guidance platform and capsule^36^. The project demonstrated impressive organ visibility rates (73–98%) and technological success rates (98%). These systems generally integrate one or more miniature permanent magnets within the capsule, controlled and propelled by the magnetic field generated by external large magnets or coils. During procedures, patients lay on a magnetically controlled surgical table, with physicians viewing the transmitted images on a display and steering the capsule wirelessly towards the area of interest^37–39^ (Fig. 2c). The precision and responsiveness of the external magnetic field to magnetic materials provide a solid foundation for assessing the adaptability and multifunctionality of WCE designs.

### Communication

Data transmission and reception strategies are pivotal to the intelligent operations of WCE. Relying on its exceptional power efficiency and high data rate, the WCE telemetry system can deliver a considerable volume of high-resolution images and other sensing data while keeping power consumption minimal.

WCE typically employs radio frequency (RF) for transmission. Depending on the frequency range, RF is further divided into categories such as low frequency, high frequency, ultrahigh frequency, and microwaves. While low frequency is easy to design and excels in skin layer penetration, its dependence on larger electronic components hinders the miniaturization process of WCE^40^. Commercially available WCE devices mostly employ ultrahigh frequency communication, usually around 400 MHz^41^. However, the 300 kHz channel bandwidth allowed in this frequency band creates significant challenges in providing the data rates required for transmitting high-quality, real-time video. These limitations make narrowband transmission technology increasingly insufficient to match the pace of advancements in WCE technology.

Ultra-wideband (UWB) communication, capable of achieving data transmission exceeding 100 Mb/s, substantially enhances video quality while decreasing power consumption, making it an ideal choice for burgeoning research into the wireless interfaces of WCE^42,43^. Defined as signals with a bandwidth of no less than 500 MHz, UWB operates within 3.1–10 GHz frequency bands^44^. Besides superior transmission rates, UWB ensures minimal power transmission and facilitates a compact form factor. Nonetheless, broader implementation may require higher hardware expenditure compared to narrowband transmission technology, including special UWB chips and antennas. Varying national regulations may also limit its widespread adoption. Yet, despite these challenges, UWB remains a promising area to watch in the field of WCE robotics duo to its high-speed transfer capabilities.

As established in the standard IEEE 802.15.6 of 2012, intrabody communication (IBC, also termed human body communication) is recognized, along with narrowband and UWB communication, as one of the three methods for constructing a wireless body area network^45^. IBC utilizes the human body as an electrical signal transmission medium, positioning it as a non-RF communication approach (Fig. 2d). In comparison to RF, IBC offers more benefits as it results in lower power consumption and a more compact capsule due to the elimination of power-intensive RF components and antennas^46^. In 2007, the Intromedic company released the first WCE device, MiroCam®, which utilizes IBC technology^47^. The capsule incorporates two strip gold plate electrodes on its shell to transmit electrical signals from the gastrointestinal tract to the exterior of the human body. These signals are then transmitted as electromagnetic waves directly from one pair of transmitting electrodes on the skin to the other pair of receiving electrodes, known as galvanic IBC^48^. There also exists capacitive IBC, a second coupling strategy where the electrical coupling between the transmitting and receiving electrodes forms a return path via external grounding, with most of the signal transmitted through the current loop between transmitter and receiver^49^. Although the transmission quality of capacitive IBC may be influenced by the surrounding environment owing to the unique path and transmission method, it offers non-contact, lower transmission power, and higher data rate compared to the galvanic one. Regardless of the coupling type used, IBC provides power consumption advantages in all communication modes while avoiding signal blocking caused by the human shadow effect. By limiting the communication range to a very small area on the surface of the human body, IBC minimizes the potential for interference between various networks^50^.

### Localization

Accurate discernment of a capsule’s position and direction within the gastrointestinal tract is integral for enabling doctors to pinpoint lesion locations for further diagnostics and interventions. It also provides crucial closed-loop feedback to control device movement. However, unpredictable gastrointestinal tissue movements and distance measurement errors arising from medium non-uniformity pose new challenges to WCE localization. As indicated in ref. ^51^, WCE positioning should adhere to benchmarks of less than 6 mm for absolute position error, and less than 5° for absolute directional error. Currently, several research teams are exploring methods, including RF localization, magnetic localization, video localization, and hybrid localization, to meet these specifications.

The RF signal wirelessly transmits distinct images from within the gastrointestinal tract to an array of body-attached sensors, and it additionally serves an instrumental role in positioning. RF localization commonly estimates position-dependent signal parameters, such as received signal strength (RSS) and time of arrival (TOA). Given its lack of identifiable health risks and minimal sensitivity to bandwidth restrictions, RSS positioning has been deployed in earlier commercial capsules like the Smartpill^TM^ ^52^ and the M2A^TM^ ^53^. Compared to RSS, TOA-based technologies attain superior accuracy by gauging inter-node distances primarily through signal arrival time measurement. It is worth noting that the high absorption property of human tissue can cause significant inaccuracies in TOA estimation, and the narrow bandwidth (402–405 MHz) of the medical implant communication service hinders high-resolution TOA estimation^54^. Implementing radio frequency identification within the gastrointestinal tract ensures lower signal latency^55^. RF localization is advantageous in terms of cost-effectiveness as it utilizes the inherent wireless communication modules in WCE devices without increasing complexity or payload capacity. Nevertheless, current literature seems to overlook the directional information of capsules when it comes to positioning solely through electromagnetic signals^56,57^.

Magnetic localization, in contrast to RF localization, benefits from transmission signals that are unaffected by dielectric and frequency-related path losses within body tissues^58^. This is due to the near-identical magnetic permeability in air, non-ferromagnetic substances, and human tissues (approximately 1). When employing magnetic localization for WCE, the customary method involves placing an axial magnetic source within the capsule. A magneto-resistive sensor array, attached either on the skin or an external surgical platform, is then used to measure the strength and direction of the magnetic field. Recent studies^59–61^ indicate that by arranging a two-dimensional or three-dimensional (3D) array of hall-effect sensors around the capsule endoscope, positioning error can be reduced to a minimum of 1 mm, with an angular error of 5.1°. Other research suggests placing magnetic sensors within the capsule and studying measurements from an external magnetic field^62,63^. Despite its less precise positioning, this method simplifies system complications caused by multiple sensors and expands external hosting space. Given its potential for integration with other intelligent technologies, magnetic localization has gained popularity in recent research. However, whether the magnetic sensors are placed inside or outside the WCE device, acquiring rotation angle information related to the internal magnetic axis remains a challenge. The magneto-mechanical resonator, as recently proposed by Gleich and colleagues^64^, introduces a special magnetic positioning method capable of achieving six degrees of freedom positioning, covering three-dimensional position and direction. Including two spherical permanent magnets, a fine filament, and a cylindrical shell (less than 1 mm^3^), the miniature device can be excited into oscillation by a pulsed current from the transmitter coil. The varying magnetic field resulting from the magnet oscillation induces different voltages in these coils, which serve for positioning after amplification. With its miniaturized size, high-precision tracking, and real-time functioning, this device is an optimal choice for tracking the position of internal medical instruments. However, its usage in WCE containing ferromagnetic or metallic components, such as antennas and receiving coils, may substantially affect positional accuracy and compatibility with subsequent intelligent technologies.

Progress in AI algorithms has bolstered the potential of video localization. This is achieved by utilizing the dynamic distortions, curls, and shape alterations observed in visual images of the gastrointestinal tract. Unlike other methods, video localization does not require additional internal or external modules to enhance positioning information transmission—it relies solely on the analysis of original video frames. Video localization can be classified into two primary categories: topographical video segmentation and motion estimation^65^. Topographical video segmentation leverages various image characteristics such as color, texture, and movement to divide filmed frames into multiple successive organ area segments, thereby facilitating accurate positioning^66–68^. On the other hand, motion estimation, based on visual odometry (VO)^69^, calculates the precise position by examining the point-feature alteration relationship between successive frames captured by the capsule’s internal image sensor. This approach to WCE localization was first proposed by Iakovidis et al. and employed a Java video analysis framework that expedited the development of intelligent video analysis applications^70^. Subsequent advancements incorporated artificial neural networks to enhance the VO method, improve geometric inferences, and augment positioning precision^71,72^. Besides determining the capsule’s position, VO can also infer directional information by estimating displacements and rotations of interest points within consecutive frames^73^. However, solely relying on video positioning may not fulfill the accuracy requirements for WCE localization, and the video transmission’s low frame rate, along with the speed of image recognition, may lead to considerable latency.

Aiming for a complementary advantage, researchers have endeavored to integrate video positioning with other positioning technologies, including magnet/video^74,75^ (Fig. 2e) and RF/video hybrid positioning^76,77^, without incorporating additional sensor components. Such hybrid localization technologies potentially facilitate high-precision positioning in next-generation WCE robots. This integration garners added information like rotational speed around the capsule’s central axis, thereby enhancing overall positioning accuracy.

### Visual detection of lesion tissue

Video detection remains a key diagnostic tool for screening small intestinal diseases. Throughout its journey within the gastrointestinal tract, a capsule endoscope routinely generates beyond 60,000 images^78^. Professional physicians have the task of examining these images to identify signs of inflammation or disease, a task that could take one to two hours. Furthermore, the overlooked detection rate for certain lesions, such as tumors, reached a staggering 18.9%^79^. Concurrently, there is a noticeable lack of consistency between experienced practitioners and novices when defining the criteria for a WCE re-examination based on the number and size of lesions^80^.

Improving image quality through processing video signals (such as lesion contours)^81^ or integrating depth information for 3D video reconstruction^82^ substantially reduces video scanning time and missed detection rates. Although this approach simplifies the diagnostic process and enhances the accuracy of detection for specific ailments, the results from human video inspection are still barely satisfactory.

The advent of AI has notably enhanced software capabilities, particularly in medical imaging analysis. AI-driven inspections autonomously interpret images, reducing clinical doctors’ workload, and producing objective and consistent results. AI algorithms, such as “express view“^83^ and “suspected blood indicator“^84^, tailored for WCE, are now utilized in clinical practice. AI uses extensive image data training sets, calculating weights based on attributes like texture, color, and shape to categorize lesions. The research of AI video inspections in WCE extends to the diagnosis of different tissues, encompassing but not limited to tumors^85^, polyps^86^, gastrointestinal bleeding^87^, and ulcers^88^ (Fig. 2f). Preliminary data suggest that autonomous detection of these conditions allows AI algorithms to achieve over 95% sensitivity, specificity, and accuracy^89^. AI enables WCE video inspections to attain high repeatability and scalability, significantly reducing costs by eliminating the need for extensive training programs.

### Diagnostic and therapeutic functions

Further advancements in the bio-MEMS field have surpassed merely visually identifying potential lesions. It has now become possible to integrate microsensors and micromechanical systems within wireless capsules. This progression facilitates superior capabilities for tissue diagnosis and treatment.

Typical image sensors in WCE are generally restricted to visible wavelengths, confining to non-specific alterations diagnosis in the mucosal surface. Addressing this, researchers introduced a variety of supplementary imaging techniques such as ultrasound^90^, X-ray^91^, and optical coherence tomography^92^, which enable deeper imaging of submucosal tissues. Assessing changes in physical environments and chemical/biological components within the gastrointestinal tract is also crucial, as they can offer indirect indicators of potential digestive diseases. For instance, Li et al.^93^ designed a capsule endoscope with two orthogonal pressure sensors to measure contact pressure between the capsule and small intestine, assisting in the detection of potential obstructions. Temperature-sensing capsules primarily monitor athletes’ core body temperatures during training^94^. Also, pH capsules like Medtronic’s Bravo^TM^ pH are utilized for monitoring gastroesophageal reflux disease^95^, and biochemical sensors can detect active bleeding^96^. Some sensors are capable of detecting intestinal gas composition, enhancing colon health^97^. Although incorporating these innovative sensors in WCE has widened the diagnostic scope, understanding the full clinical impact of these indirect methodologies on the gastrointestinal tract and necessary subsequent therapeutic measures remain at issue.

Enhancing WCE capabilities is fundamentally associated with its intricate microstructure design. The strategic positioning of diagnostic and therapeutic components within its limited volume is currently a significant focal point of interest within the scientific community. Recent advancements in manufacturing procedures have allowed capsules to include tools such as biopsy needles, scrapers, and micro forceps, enabling biopsy sampling from beneath the mucosal tissues^98–100^. Innovative scholars have introduced the first therapeutic WCE device capable of unleashing clamps, paving the way for treating gastrointestinal bleeding and facilitating the closure of internal incisions^101^. Moreover, the capsule’s ability for targeted drug delivery has been clinically beneficial. A notable example is the widely adopted “Enterion” capsule^102^, engineered to deliver a diverse array of drug formulations, including solutions, powders, and particles, to any desired region within the intestine.

Previous studies have highlighted magnetic field propulsion as an advantageous alternative^99,100,102,103^. It operates via the rapid rotation or linear movement of the permanent magnet inside the capsule, instigated by an external magnetic field, which in turn facilitates treatment (Fig. 2g). Therefore, the following section will focus on explaining the progression of magnetic-controlled diagnosis and treatment in WCE.

## Advanced intelligent technologies

This section presents a review of the latest findings from six cutting-edge intelligent technologies in the WCE field for the period 2018–2023. It also includes an analysis of the challenges faced and the corresponding solutions implemented, as shown in Table 1. It should be particularly noted that some wireless capsule robots may lack imaging capabilities duo to simplified design schemes and differences in research focus, they are still classified as WCE robots and are thus within the scope of discussion.

**Table 1:** Key challenges and solutions for WCE advanced intelligent technology

Technology	Challenge	Solution	Refs.
Near-field WPT	Low power transmission efficiency	Enhance the quality factor of the transmitting coil	^105^
		Decrease the load factor	^106^
	Poor received power stability	Ensure the uniformity of the magnetic field generated by the transmitting coil	^107–110^
		Three-dimensional receiving coils	^111,112^
Magnetic field active drive	Tissue damage and intestinal torsion	Reciprocally rotating magnetic actuation	^118^
	Locomotive performance improvement of WCE	Three-dimensional Helmholtz coils	^120–122^
	Low magnetic field intensity	Incorporates an iron core	^99,124^
	The patient’s tolerance in the supine position	Saddle and rectangular coil drive systems	^39,125,126^
UWB/IBC	Economizes on the internal space within the WCE device	Conformal UWB antennas	^129^
	Polarization mismatch and multipath fading	Dual-polarization antennas	^43^
	Bidirectional IBC	Combination between RF and IBC	^130,131^
	Drug release monitoring	Proteus Discover, IBC coupling mode switching	^135,136^
Hybrid location	Positioning accuracy improvement	Incorporates both magnetic and visual elements	^75^
		A hybrid RF with vision aware fusion scheme	^137^
AI-based autonomous lesion detection	Acute gastrointestinal bleeding	Simplified machine learning algorithms	^144^
		The bleeding image recognizer	^145^
	More detailed classification based on the degree of polyp canceration	CNN architectures: D-Caps	^147^
	Ulcer detection	CNN architectures: AlexNet and GoogLeNet	^148^
	Multilesion detection and classification	Combination of baseline model and transfer learning strategy	^149^
Magnetic control for biopsy/drug delivery	Integrated micro biopsy device	Fine needle capillary biopsy	^150,151^
		Tissue biopsy scraper	^152,153^
	Multi-point targeted liquid sampling	External precessional magnetic field	^158^
	Passive and active drug delivery control	Internal magnet, magnetic conductor, or magnetic fluid	^159–162^

*WPT* wireless power transmission, *UWB* ultra-wideband, *IBC* intrabody communication, *CNN* convolutional neural network.

### Near-field WPT

The principal difficulties in applying near-field WPT technology to WCE include deficient power transmission efficiency (PTE) and poor received power stability (RPS). A large portion of the research revolves around achieving high-efficiency and high-stability in WPT while prioritizing human safety and comfort.

For weakly-coupled WPT systems, the PTE of WCE can be defined as^104^

$${{{\rm{PTE}}}}=\frac{\alpha * {k}^{2}{Q}_{{TC}}{Q}_{{RC}}}{(1+{\alpha }^{2})}$$

where *k* is the coupling coefficient, ${Q}_{{TC}}$ and ${Q}_{{RC}}$ are the quality factors of TC and RC respectively, and $\alpha$ is the load factor. The parameter *k* illustrates the extent to which the changing magnetic flux triggered by the transmission coil, is successfully captured by the RC. This interaction largely depends on the relative positioning and dimensions of the two coils. However, it is difficult to improve PTE and RPS by increasing the value of *k* in the actual application of WCE. The only viable strategy might be to enhance ${Q}_{{TC}}$ and ${Q}_{{RC}}$ or decrease $\alpha$.

$Q$ characterizes the resonance capability of resonant circuits. Sekiya et al. implemented high ${Q}_{{TC}}$ coils, which were constructed from double-sided, high-temperature superconducting coated conductors^105^. This implementation not only boosts the system’s PTE but also enhances its resilience to axial and angular misalignments when contrasted with copper-based TCs.

$\alpha$ signifies the ratio of load resistance to the effective series resistance in the RC. Most existing studies have merely demonstrated the WPT capability of WCE in free space, failing to explore its functionality in authentic work environments, thus overlooking the impact of $\alpha$. Miah et al.^106^. Evaluated the performance of their proposed WPT system by situating their receiving coil in both hollow and colonic liquid-filled models. Their approach first involved optimizing load impedance before matching it to the actual capsule load using impedance conversion technology, resulting in a noteworthy improvement in PTE.

Optimal RPS hinges on the uniformity of the magnetic field generated by the TC. A standard setup frequently considered is the Helmholtz coil (HC) pair, available in both circular and square shapes, known for generating a uniform magnetic field. Existing computational models enable calculations of electromagnetic field uniform intensity for both circular HC and square HC^107^. Results show that the square HC outperforms the circular ones in uniformity and also conserves energy. Besides HC, other variations of coil pairs can generate sufficient uniformity. Zhuang et al. proposed an optimized, space-efficient design using a plane square spiral coil pair (PSSCP) as the TC^108^. The PSSCP creates a steady magnetic field, and it also flexibly adjusts the space between the coils according to the patient’s requirements, leading to fewer limitations imposed by the electromagnetic coils. An innovative WPT system featuring two parallel opposed coils was suggested^109^. This arrangement incorporates a magnet core within the TC for the first time, substantially improving both PTE and RPS while retaining magnetic field uniformity. Furthermore, increasing the number of coils represents an alternative method for generating a uniform magnetic field^110^.

The uncertainty in capsule orientation restricts the ability of a one-dimensional, single-coil RC to consistently deliver stable power to the capsule. In addressing this issue, Khan et al. proposed an innovative design that implements a flexible dual TC and a cross-shaped, 3D RC with a diameter of 8mm^111^. When combined with a nested multidimensional optimization algorithm, this arrangement improves portability, patient comfort, and the capability to manage variations in capsule angles. Additionally, a ferrite-encapsulated 3D-RC is specifically designed to meet crucial parameters like tolerance for capsule misalignment, compactness, adequate heat dissipation, and satisfactory PTE^112^.

### Magnetic field active drive

Linear and rotational movements in the capsule robot can be induced by applying magnetic forces and torques to the permanent magnets inside the capsule. The literature^113^ presented a handheld external magnetic field navigator (MFN) for WCE, which empowers endoscope operators with the ability to remotely moderate the capsule’s motion and viewing area. Though cost-effective and intuitively operated, manual magnetic manipulation could encounter issues with precision and repeatability, potentially leading to difficulties for skilled operators. A substitution for MFN, in method, is a robotic arm that eliminates the need for human intervention, providing a more accurate control of capsule endoscopy. It has become a preferred option in the current commercial sector for magnetically controlling capsule endoscopy^114^. In a study conducted by Ciuti et al.^115^, a robot was employed to regulate the permanent magnet controlling the capsule motion. Their research constituted the first demonstration where robot-assisted control surpassed manual control concerning target accomplishment efficiency.

**Fig. 3:**
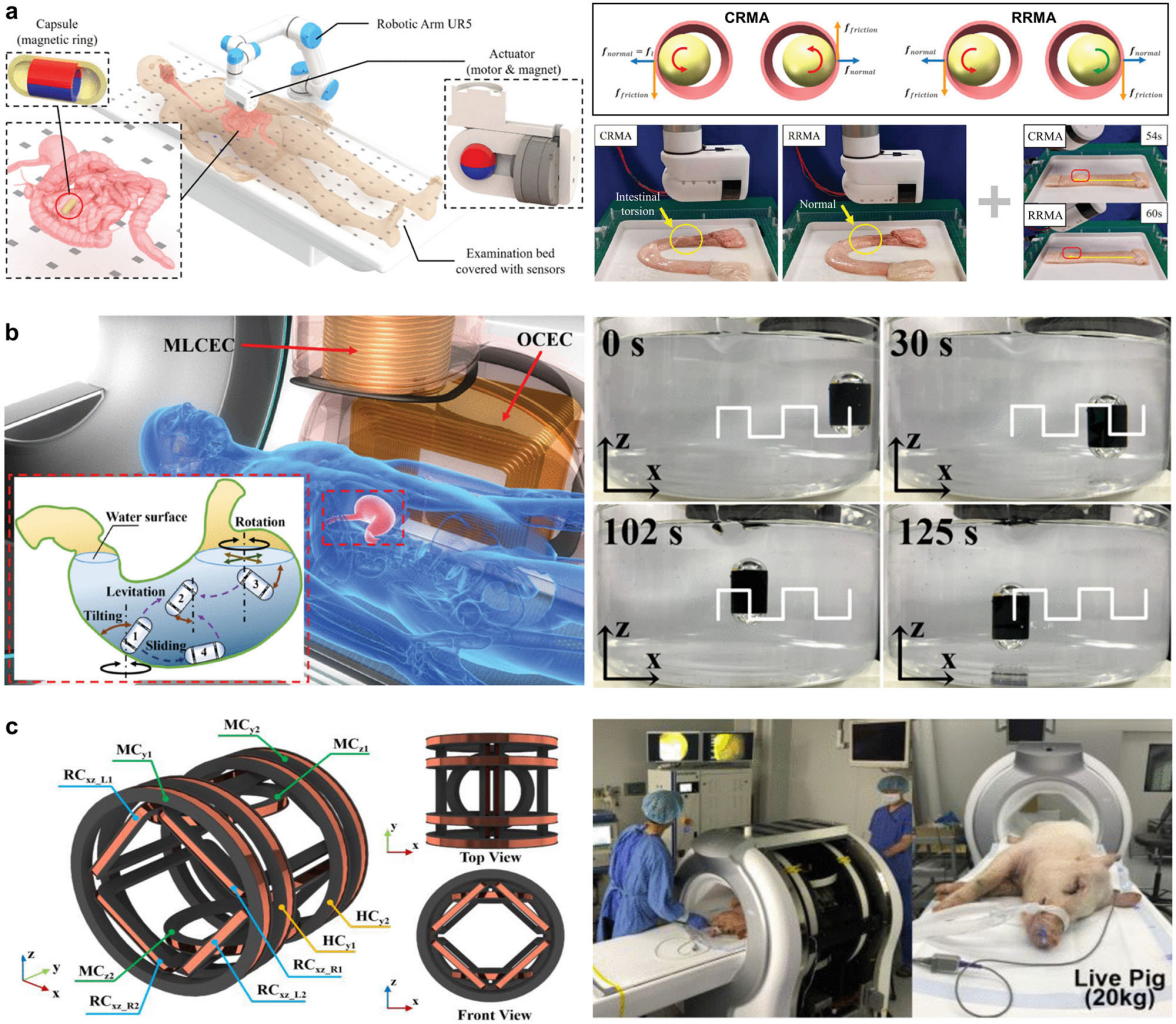
Magnetic field active drive of WCE. **a** An external magnet drives the WCE robot safely through the intestine with RRMA. Left: schematic representation of the platform. Top right: force analysis. Bottom right: comparative experiments of intestinal capsule movement. CRMA continuous rotation magnetic actuation, RRMA reciprocally rotating magnetic actuation. **b** The added electromagnetic coil realizes the high-precision levitation control of the capsule robot. Left: schematic representation of the platform. Right: an image sequence of the capsule model performing magnetic levitating translational motion at a desired square wave trajectory. MLCEC magnetic levitation control electromagnetic coil, OCEC orientation control electromagnetic coil. **c** The rectangular coil drive system increases the tolerability of patients in decubitus position and has been tested in pig clinical trials. Left: the electromagnetic coil model. Right: in vivo experimental setup using a live pig. HC Helmholtz coil, MC Maxwell coil, RC rectangular coil. Panels reproduced with permission from: **a** ref. ^118^, IEEE; **b** ref. ^121^, IEEE; **c** ref. ^39^, IEEE.

When employing an external permanent magnet to propel a WCE robot through the intestine, there are generally two methods: direct drag based on magnetic force and continuous rotation magnetic actuation (CRMA) based on magnetic torque. The former is simpler to implement but easily results in high pressure and friction between the capsule and intestinal wall due to the close proximity of internal and external magnets, potentially causing tissue damage^116^. On the other hand, CRMA involves a continuously rotating external spherical magnet to generate a rotating magnetic field, driving the externally threaded capsule forward in a spiraling motion^117^. However, this approach also presents safety concerns, such as the risk of intestinal torsion in the periodically contracting small intestine environment. A recent study by Xu et al.^118^ introduced reciprocally rotating magnetic actuation (RRMA) which utilizes an external rotating magnet and a smooth capsule to effectively mitigate these risks (Fig. 3a). By frequently changing the rotation direction of the magnet to stretch and open the intestines, their experiments demonstrated that RRMA significantly reduced environmental resistance while ensuring intestinal safety, thus improving driving efficiency within confined tube environments.

In contrast to the movable external magnet system, the electromagnetic coil system significantly improves flexibility in controlling the capsule robot’s magnetic field, encompassing modulation of field power and magnetic field toggling^119^. Integrating core elements like electromagnets, HCs, Maxwell coils, and saddle coils, the electromagnetic coil system influences the intensity and orientation of the resultant compound magnetic field by regulating the current flowing through each coil.

Utilizing a 3D HC alongside the capsule’s rotary screw jet configuration bolsters its locomotive performance^120^. An innovative electromagnetic coil system premised on adaptive magnetic levitation is proposed to enhance the control over the floating motion of the capsule^121^ (Fig. 3b). This system incorporates a coil with three degrees of freedom underpinning the 3D HC, designed to rectify the constraints of the preceding coil-based magnetic levitation system and to adjust the capsule’s tilt angle. In addition, a coil arrangement, including a 3D HC and one-dimensional Maxwell coils, validates the robot’s ability to execute the helical advance movement in a glass tube and two-dimensional planar movement in a stomach model^122^.

The magnetic field intensity generated by the coil may not fulfill the capsule’s propulsion needs, particularly in expansive workspaces. According to magnetic field theory^123^, there is a direct proportionality between the magnetic conductivity and the coil’s generated magnetic field strength. Consequently, incorporating an iron core (denoted electromagnet) into the design markedly bolsters the magnetic induction strength. In 2010, Kummer et al. conceptualized OctoMag, an electromagnetic propulsion system constituted of four pairs of electromagnets^124^. The system’s unique characteristic lies in its control mechanism, which uses intricate non-uniform magnetic fields, specifically, the joined field created by several cooperative soft magnetic core electromagnets. Son et al.^99^ integrated an additional coil into OctoMag to generate a more powerful magnetic field gradient whilst significantly reducing power wastage through redundancy.

When designing the electromagnetic coil system, patient tolerance in the supine position must be factored in. The saddle coil scheme, first employed by Jong-Oh Park and his team at Chonnam National University for an active drive on WCE robots^125^, serves as a more suitable option. They developed an active gastrointestinal WCE system, “ALICE”, comprising five sets of coils: two pairs of saddle coils and one HC pair for alignment, coupled with an additional pair of saddle and Maxwell coils for propulsion. This division of alignment and propulsion coils simplifies the facilitation of complex movement diagnoses. The team further engineered two rectangular coil drive systems, the MACE^126^, and EMS^39^ (Fig. 3c). This sophisticated electromagnetic field control setup enhances magnetic force, reduces peak input current, and as a result, diminishes performance deterioration. In comparison to previous configurations, this design significantly elevates the magnetic field intensity, ensures lower input current, and prolongs operational periods by mitigating heat effects.

### UWB/IBC

Serving as an integral component, the antenna establishes a vital communication link between the capsule and the exterior receiver. For UWB communication to be effective, the antenna must consistently exhibit uniform gain and radiation properties across the full UWB band, demonstrating resilience to frequency shifts. Initially, antenna design schemas were entirely planar, delivering commendable data transmission efficiency in conjunction with omnidirectional radiation^127,128^. However, given the necessity to optimize internal space within the WCE device, the creation of a conformal UWB antenna has become a crucial requirement. Shang and Yu suggested a symmetrical double-loop antenna boasting ultra-wideband features^129^. Upon experimental deployment within pork, this particular model produced a measured bandwidth of 124%.

Mitigating issues related to polarization mismatch and multipath fading in wireless body area network communication systems preferentially requires the use of dual-polarization antennas over linear polarization ones. Li et al.^43^ pioneered the examination of UWB dual-polarization antennas specifically for WCE applications. Given the antenna’s broad frequency band coverage, high isolation, and distinct UWB properties, it emerges as a promising candidate for communications within industrial, scientific, and medical systems.

For IBC, recent research majorly focuses on achieving bidirectional communication and drug release monitoring.

Market-available WCE devices chiefly deploy a unidirectional communication system that exclusively transmits images from the capsule. An evident drawback of this system is its inability to adjust the capsule’s operation concerning the targeted research area pre-surgery. To address this IBC limitation that only enables unidirectional communication from the capsule to an external receiver, studies^130^ and^131^ explored RF inward communication from the exterior for WCE devices and its cohabitation with IBC. The telemetry signal, externally modulated using passive RFID, is then conveyed to the capsule at a frequency of 13.56 MHz. Notably, this approach enables bidirectional communication for WCE without increasing power consumption.

Monitoring drug release by embedding edible electronics in capsules enables real-time tracking of a patient’s medication intake by physicians. This approach has been clinically verified for reducing serious complications and worsening conditions due to medication nonadherence^132–134^. In 2015, Proteus Digital Health Inc. launched the first Proteus Discover system, based on galvanic IBC, which monitors patients’ daily medication intake in a compact size (1 mm^3^) with low power consumption^135^. The miniature sensor in the system, upon contact with gastric acid, initiates an electrochemical reaction that provides sufficient energy to emit an electrical signal detectable by an external patch. As the sensor electrodes and drugs dissolve gradually over time, the electrical signal changes in conjunction. This correspondence reflects drug intake time and dosage. A recent study^136^ suggests that IBC coupling mode switching can optimize this communication process. Similar to Proteus Discover, drugs and edible electronic devices gradually dissolve in gastric acid. The process reduces the variable impedance, triggering a “short circuit” in the signal and transitioning from capacitive to galvanic IBC. Given that the signal transmission attenuation of galvanic IBC is higher than the capacitive one, this switching results in a reduction of 96.5% of the read-out signal, ultimately improving the detection limit of edible pills for monitoring drug absorption and achieving complete drug absorption detection.

### Hybrid location

In the section “Localization”, we discussed the pros and cons associated with three distinct methods: RF localization, magnetic localization, and video localization. We concluded that the most effective positioning strategy involves integrating two of these technologies. An extensive literature review in the database, spotlighting hybrid positioning methodologies in WCE over the past five years, primarily emphasizes the following two articles.

In literature^75^, researchers introduced a hybrid positioning method known as “MagnetOFuse”, which integrates both magnetic and visual aspects. The proposed technique employs a capsule equipped with four sidewall cameras and a magnet. Magnetic localization is accomplished using nine triaxial Hall effect sensors to ascertain the capsule’s global positioning. However, relying solely on magnetic positioning does not distinguish between the capsule and tissue movements. To overcome this issue and improve tracking accuracy, sidewall cameras are deployed to observe the capsule’s motion, rather than the spontaneous movements of the gastrointestinal system. The study demonstrated that the average location error of this hybrid approach is a minimal 3.5 mm, effectively offsetting the relative movements of the gastrointestinal tract.

Literature^137^ proposed a combined positioning method, referred to as the “RF with vision aware fusion” (RF-VaF) scheme. RF-VaF comprises three modules: an RF-based positioning module, a vision-based positioning module, and a fusion-based positioning module. In RF-based positioning, a synthesis of time of flight, RSS indicator, and capsule angle via an integrated sensor array is used to infer the capsule location. Simultaneously, the vision module introduces a novel convolutional neural network (CNN) specifically for frame registration, correlated image generation, intelligent pixel feature matching, and multi-feature extraction. To calculate the precise endpoint location, the fusion module applies the hybrid computing architecture algorithm.

### AI-based autonomous lesion detection

**Fig. 4:**
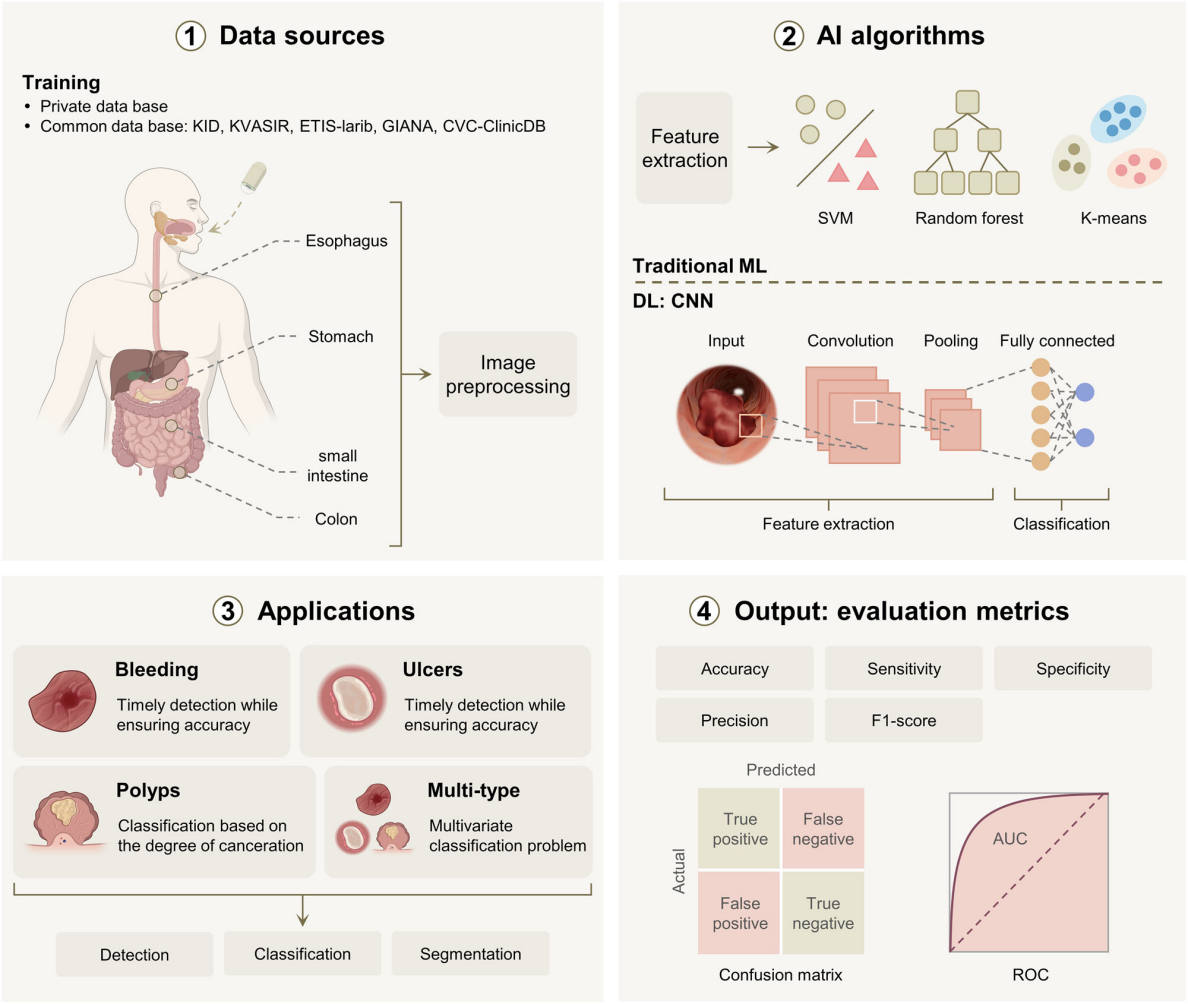
AI-based comprehensive pathological analysis model. The process begins with the WCE device capturing gastrointestinal images, which are then preprocessed and used as input for the AI algorithm. The algorithm’s architecture can be categorized into traditional machine learning and deep learning based on varying feature extraction and classification methods. Subsequent image processing is carried out in conjunction with the specific application scenarios and analysis tasks of WCE. Ultimately, the model outputs relevant metrics to determine its performance. ML machine learning, DL deep learning, SVM support vector machine. Figure 4, created with BioRender.com, released under a Creative Commons Attribution-NonCommercial-NoDerivs 4.0 International license.

The process of AI-based autonomous lesion detection can be conceptualized as a comprehensive pathological analysis model (Fig. 4), comprising four key components: data sources, algorithms, applications, and evaluation metrics. Algorithms play a vital role in evaluating the adequacy of newly developed models, particularly given the swift advancements in machine learning (ML) and deep learning (DL) within the domain of image recognition.

Before the advent of CNN, traditional ML algorithms were commonly employed for WCE image abnormality detection. For example, some studies^138,139^ focused on detecting small intestinal polyps using the support vector machine (SVM). SVM leverages features like polyp texture or size as decision boundaries, also referred to support vectors, to perform classification and regression tasks. Additionally, other methodologies such as random forest^140^ and K-means clustering^141^ were also involved. ML typically exhibits faster training and prediction speeds compared to DL when dealing with smaller datasets. Due to advantages in computational resources and model interpretability, ML continues to hold a significant position in AI image detection, even in the era of big data.

CNN is a mainstream DL algorithm known for its exceptional feature extraction and classification capabilities in the field of medical image recognition^142^. It is typically composed of four elements: an input layer, a convolutional layer, a pooling layer, and a fully connected layer^143^. Initially, the gastrointestinal tissue image traverses through the convolutional layer, where each kernel executes a convolution operation on the image, thereby constructing a set of feature maps. Subsequently, the feature maps’ spatial dimensions get reduced by the pooling layer, thus diminishing computational complexity and mitigating overfitting. Lastly, the fully connected layer transforms these high-level feature maps into outputs for lesion classification. Notable CNN variants like AlexNet and VGG have gained popularity in recent years, showcasing advancements built upon the CNN framework. Alongside CNN, the multi-layer perceptron (MLP), a type of artificial neural network, is often utilized for wound detection in resource-constrained environments due to its simplicity and effectiveness in handling nonlinear problems^144^.

Considering the benefits of CNNs for imaging detection and limited space, we discuss recent key studies in lesion detection utilizing CNNs. A more comprehensive comparison of all pathology recognition models developed in the past five years can be seen in Supplementary Table 2.

Present research prioritizes the autonomous detection of acute gastrointestinal bleeding, where swift identification is crucial to prevent complications. By capitalizing on simplified machine learning algorithms for image classification, identification times can be efficaciously shortened without compromising detection accuracy. Hajabdollahi et al.^144^ have demonstrated the effectiveness of this approach by employing a simplified multilayer perceptron and CNN respectively, achieving a significant decrease in computation operations with an AUC (defined as the area under the ROC curve) exceeding 0.97. Additionally, detection precision within bleeding area frames has been improved with a novel model - the bleeding image recognizer (BIR)^145^. This model integrates MobileNet with a custom CNN model for bleeding image classification, utilizing MobileNet’s basic computational capacity before feeding its output to CNN for in-depth processing. The BIR model achieved an accuracy of 0.978 when assessed with Google’s WCE image dataset, surpassing the performance of existing methods.

During routine colonoscopy examinations, swift identification and analysis of polyps is crucial for determining their malignancy and preventing severe diseases like colorectal cancer. This process can be broken down into two main components: identifying the presence of polyps and further classifying them based on the degree of canceration. In the field of polyp detection, Nadimi et al.^146^ explored the detection of colorectal polyps with an enhanced ZF-net model. By integrating transfer learning, preprocessing, and data augmentation, the CNN algorithm achieved an impressive accuracy rate of 98%. Furthermore, a novel capsule network architecture (D-Caps) was introduced to enhance the feasibility of optical biopsy for colorectal polyps in WCE^147^. D-Caps incorporate innovative features like local constrained routing and capsule average pooling, resulting in an accuracy rate of 82% for distinguishing between proliferative polyps and adenomatous polyps.

For ulcer detection, both AlexNet and GoogLeNet have been extensively evaluated as highly accurate identification solutions in object classification, despite being time-consuming^148^. These two prominent CNN architectures were pretrained on a subset of the ImageNet database to identify the best combination of network parameters, achieving zero classification errors.

In addition, lesions in the gastrointestinal tract come in various forms, and focusing on just one of them is not enough. A recent study introduced a multipathology system for WCE images, aiming to detect and classify different lesions within the digestive system^149^. This system combined a baseline model and transfer learning strategy to realize the image classification of polyps, ulcerative colitis, esophagitis, and a healthy colon.

### Magnetic-controlled diagnosis and treatment

**Fig. 5:**
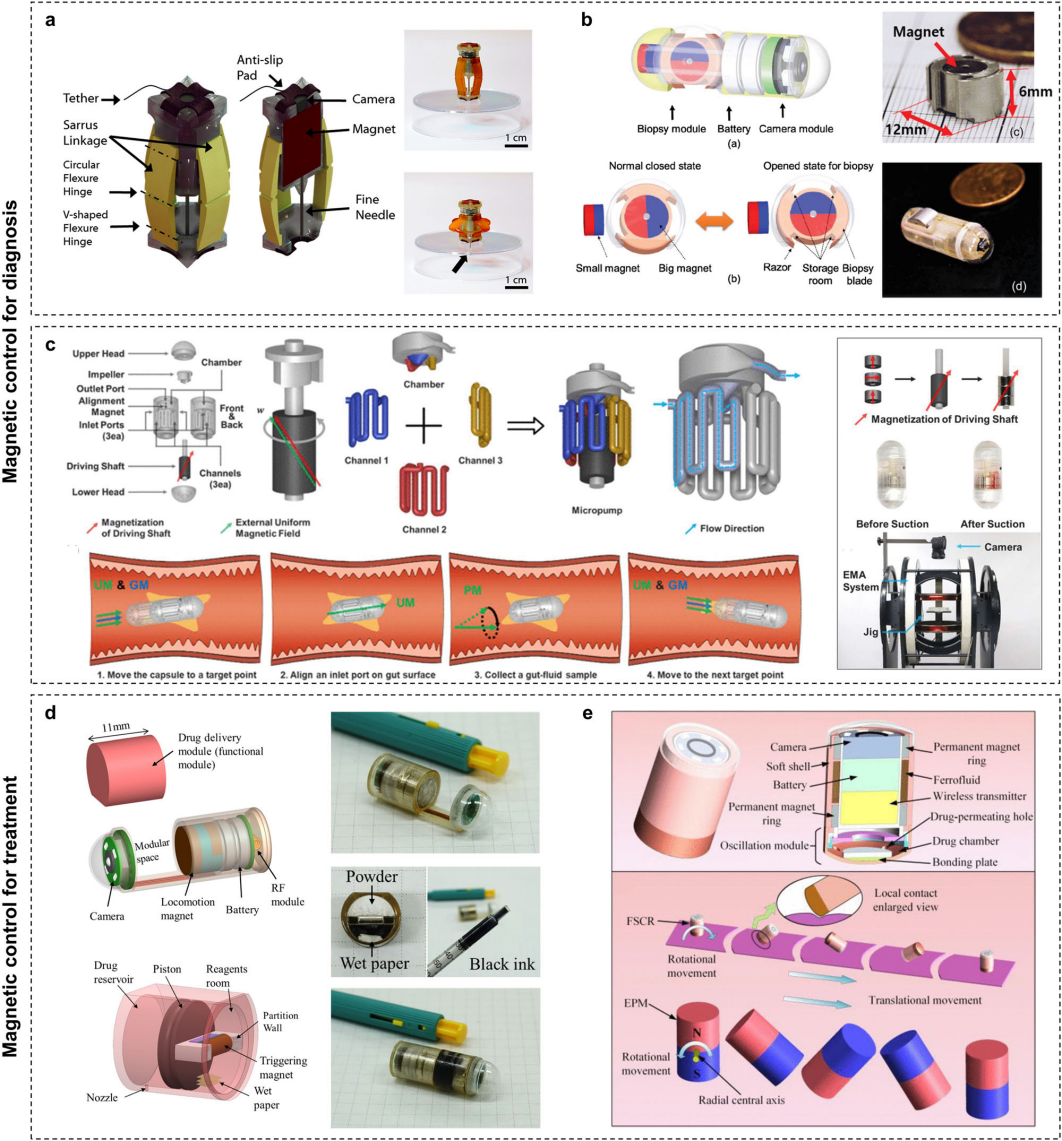
Magnetic control capsule robots for diagnosis and treatment. **a** Micro-needle capillary biopsy capsule robot based on soft Sarrus linkage concept. Left: three-dimensional model design. Right: actual deformation of the robot under magnetic force. **b** Biopsy capsule robot with scraper assembly. Top left: conceptual design of WCE robot with biopsy module. Bottom left: the proposed biopsy module and its states during locomotion and biopsy operation procedure. Top right: assembled biopsy module. Bottom right: completed prototype of WCE device for biopsy. **c** Active multiple-sampling capsule for gut microbiome. Top left: overall design. Bottom left: sampling process of the capsule controlled by an external magnetic field. Right: fabricated components and a fully assembled capsule. UM uniform magnetic field, GM gradient magnetic field, PM precessional magnetic field. **d** Passive magnetic drug delivery capsule. Top left: design of drug delivery capsule endoscope. Bottom left: structure of proposed drug delivery module. Right: assembly and prototype of the WCE. **e** Active magnetic drug delivery capsule. Top: exterior and section view of the FSCR. Bottom: surface rolling locomotion diagram of the FSCR and EPM. FSCR ferrofluid soft capsule robot. Panels reproduced with permission from: **a** ref. ^150^, Mary Ann Liebert, Inc.; **b** ref. ^152^, IEEE; **c** ref. ^158^, IEEE; **d** ref. ^159^, Springer Nature; **e** ref. ^162^, IEEE.

Fine needle aspiration biopsy and fine needle capillary biopsy (FNCB) are standard procedures for submucosal tissue sampling during endoscopic processes. However, these methods remain unexplored in WCE. Leveraging magnetic field drive and soft robotics technology, Son et al.^150^ demonstrated a capsule robot capable of performing FNCB (Fig. 5a). The device’s design encompasses principles from the soft Sarrus linkage concept and miniature permanent magnets, facilitating reciprocating motion influenced by a magnetic field from an external electromagnet array, thereby enabling multiple biopsies. Hoang et al.^151^ similarly proposed a screw motion FNCB mechanism that converts an internal permanent magnet’s rotational movement into linear motion within the rotating magnetic field, preventing unnecessary tissue extraction and potential perforation.

The implementation of a scraper for tissue biopsies has been verified. As detailed in the literatures^152,153^, the capsule robot integrates four synchronously manipulated scrapers by internal magnets (Fig. 5b). Magnetic attraction autonomously locks the scraper in its initial state. During tissue scraping operations, an externally generated magnetic field can overpower this attraction.

Except for tissue biopsy, it is equally important to consider liquid sampling within the gastrointestinal tract and subsequent microbiome analysis for diagnosing digestive diseases. Previous research mainly focused on analyzing excreted feces to study gut flora and metabolites, which could show significant differences^154^. Recently scholars have delved into fluid sampling at physiologically relevant sites within the gastrointestinal tract using a magnetic-controlled capsule^155–158^. These WCE devices enable fixed-point microbial capture, sealing, and safe transportation of samples to prevent cross-contamination. In a particular case^158^, an active multiple-sampling capsule facilitated the collection of various intestinal fluid samples at specific locations using three channels and a diagonally magnetized magnet (Fig. 5c). By aligning the channel entrance with the target location and applying an external precessional magnetic field, the magnet acts as an impeller for a centrifugal micropump, enabling independent rotation and sampling. The innovative multi-point targeted sampling approach suggests that WCE robots could potentially revolutionize gastrointestinal diagnosis.

Harnessing magnetic fields in drug delivery employs both passive and active approaches. In passive utilization, the magnetic fields solely trigger the drug release mechanism. For instance, Nguyen et al.^159^ used the propulsive force generated by tiny magnets within capsules in a gradient magnetic field to trigger a chemical reaction, thereby creating the necessary pressure for drug ejection (Fig. 5d). This pressure stemmed from the carbon dioxide gas produced during the reaction. Guo et al. suggested a new fixed-point drug delivery structure based on a swarm of multicapsule robots filled with magnetic fluids^160^. The magnetic fluid within the WCE robot can be magnetized by an external magnetic field, producing a clamping force between two capsules and leading to drug encapsulation. Disengagement of the magnetic field leads to the immediate disappearance of the magnetic fluid’s residual magnetism, thereby initiating drug release.

In terms of active drug delivery, the magnetic field directly induces drug expulsion. This can be achieved by augmenting the quantity of internal magnets^161^. Utilizing magnets of varied volumes can form a composite magnetic moment, which enables rotational propelling in a relatively low-strength external magnetic field. When exposed to a stronger directional magnetic field, synchronous alignment with the magnetic field direction occurs, leading to repulsion that triggers drug release. Another alternate involves the oscillatory interactions between the magnet and the conductive magnetic ring^162^ (Fig. 5e). Similar to a pump’s squeezing mechanism, this oscillation is driven by the reciprocating axial motion of the external magnet. It is worth noting in this article that the magnetic circuit is improved through the combination of the ferrofluid and permanent magnet rings, making the locomotion of the device more controllable and secure.

## Translational strategies for clinical integration

**Fig. 6:**
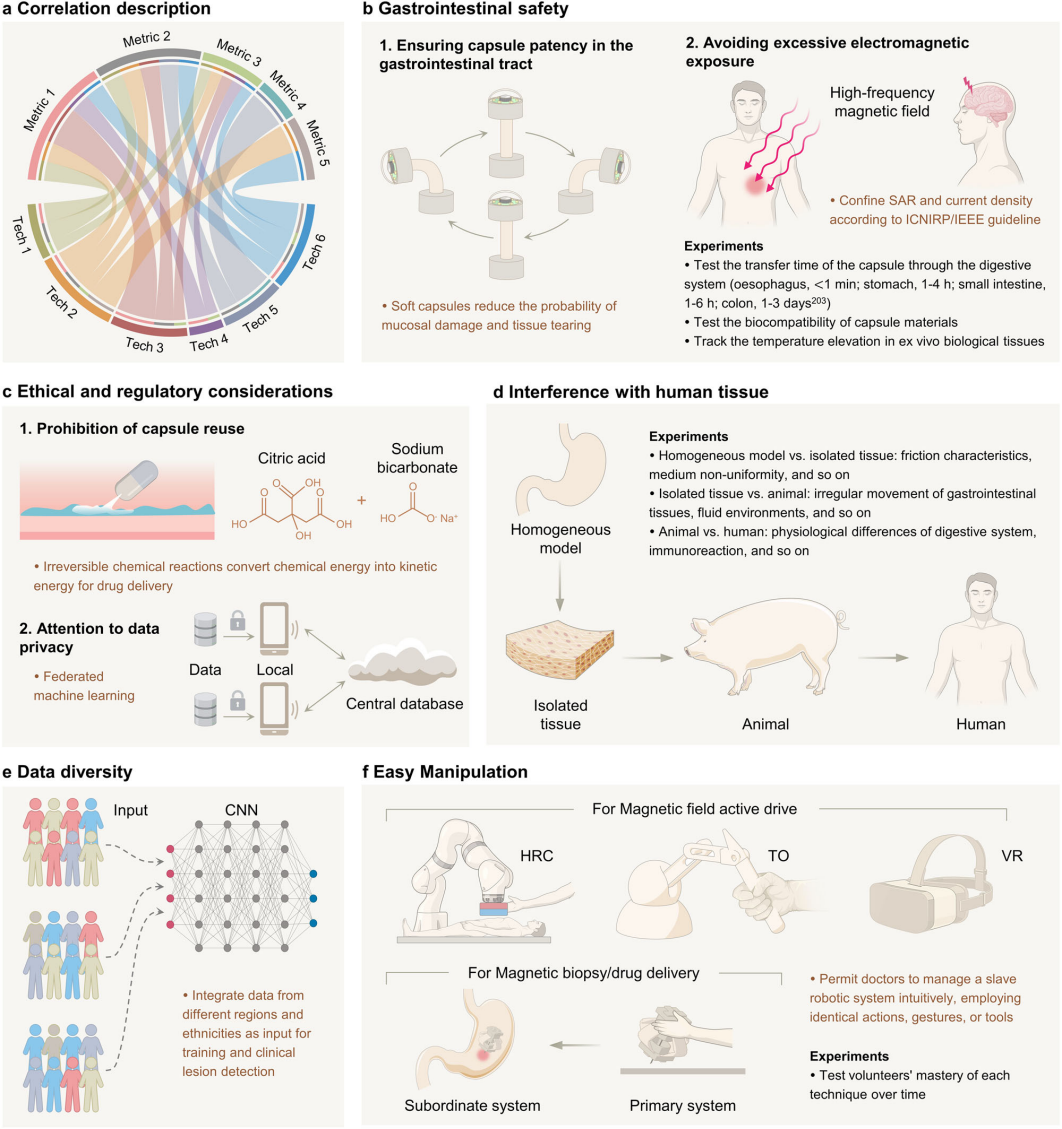
Translational strategies of WCE for clinical integration. **a** Correlation between WCE clinical indicators and advanced intelligent technologies. Metric 1–5: from gastrointestinal safety to easy manipulation; Tech 1–6: from near-field wireless power transmission to magnetic-controlled diagnosis and treatment. **b**–**f** Guidelines and key experiments. **b** Gastrointestinal safety^203^. SAR specific absorption rate, ICNIRP Non-Ionizing Radiation Protection, IEEE Electrical and Electronics Engineers. **c** Ethical and regulatory considerations. **d** Interference with human tissue. **e** Data diversity. **f** Easy manipulation. HRC human-robot collaboration, TO teleoperation, VR virtual reality. Individual images adapted with permission from: **b** ref. ^165^, Wiley; **c** ref. ^203^, Springer Nature; **f** ref. ^150^, Mary Ann Liebert, Inc. Figure 6, created with BioRender.com, released under a Creative Commons Attribution-NonCommercial-NoDerivs 4.0 International license.

Novel medical devices must undergo a series of clinical trial evaluations from their theoretical proposal to market application. When it comes to the clinical integration of intelligent WCE devices, it demands not only performance advances but also significant emphasis on safety and ethical considerations. This is crucial to meet the certification standards established by international regulatory bodies like the EMA or FDA for category III medical device registration^163^. To facilitate academia and clinicians in maximizing the development of “capsule surgeons”, we present specific clinical translation strategies for the cutting-edge technologies of WCE. This guideline covers the assessment of five common metrics and key experiments (Fig. 6).

### Gastrointestinal safety

#### Ensuring capsule patency in the gastrointestinal tract

Usually, the integration of new technologies into the capsule compromises its original shape or size. And direct clinical application could lead to symptoms like intestinal obstruction and perforation. One potential solution is to pre-swallow a patency capsule of identical specification to evaluate intestinal smoothness, thereby reducing retention risk^164^. Concerning UWB communication, as previously described, a specialized conformal antenna can maintain the capsule’s original shape while occupying less space^129^. Additionally, a novel approach proposed in the literature^165^ suggests using a magnetically responsive polymer soft shell instead of the traditional hard shell and permanent magnet. This soft design reduces the probability of mucosal damage and tissue tearing without generating harmful pressure. The same structure can also be applied to magnetically controlled biopsy capsules to promote deformation recovery and prevent non-target tissue damage from functional components^150^. However, there is an exposure risk when applying neodymium iron boron magnetic material, which raises potential poisoning issues. Superparamagnetic iron oxide nanoparticles potentially serve as a suitable alternative material and have already been applied to numerous clinical biomedicines^166^. Furthermore, for capsules that cannot maintain a cylindrical shape initially, briefly wrapping key components with ice may be an effective strategy^167^. All of the above designs should be evaluated for patency by testing the capsule transit time through the gastrointestinal tract and conducting necessary biocompatibility tests.

#### Avoiding excessive electromagnetic exposure

In the context of high-frequency magnetic field applications, such as near-field WPT, UWB communication, and RF positioning, excessive electromagnetic radiation causes damage to human organs and impairs their functionality. Including overheating from electromagnetic energy absorption in tissues and neural stimulation caused by induced currents^168^, these impacts are respectively evaluated by the specific absorption rate (SAR) and current density. Various regulations have been established to limit the range of these two parameters at different frequencies. For instance, the International Commission on Non-Ionizing Radiation Protection (ICNIRP) has set forth the “Guidelines for limiting exposure to time-varying electric, magnetic, and electromagnetic fields (up to 300 GHz)”^169^, which is internationally recognized as the most authoritative statement. Similarly, the Std C95.1-2019^TM^ established by the Institute of Electrical and Electronics Engineers (IEEE) also adopts a standard with minor numerical discrepancies^170^. To ensure that SAR and current density remain within the prescribed limits, rigorous theoretical derivations and multi-physics field simulations are required during the design process, along with real-time monitoring in practical experiments. Given the complexity of direct measurements, a common alternative involves tracking the temperature elevation in ex vivo biological tissues^171^. In addition, some proven SAR optimization methods include increasing the gap between the human body and the coil^108^.

### Ethical and regulatory considerations

#### Prohibition of capsule reuse

Commercial capsule endoscopes primarily serve as single-use medical devices to prevent cross-infection and health risks. When wireless charging coils, magnets, and biopsy components are integrated into the capsule, the rise in its own cost potentially tempts unscrupulous vendors towards recycling and reselling. To mitigate such tendencies, we suggest two measures:

1. Design irreversible structures, especially for diagnostic and therapeutic functions. For example, capsules using a chemical-reaction trigger mechanism mostly have just a single energy conversion scope unless refilled with chemicals after dismantling^172^. This design decreases the likelihood of reutilization substantially but may lead to compromised accuracy because of restrictions on repeated drug delivery.
2. Add obvious, hard-to-remove labels to the capsules. Similar to barcodes or QR codes, these unique labels would be registered as “used” after the inaugural scan by medical professionals.

#### Attention to data privacy

There is a partial overlap between the frequency bands involved in UWB communication and the frequency ranges applied by 5G mobile communication as well as amateur radio enthusiasts^173^. The conflict might lead to leakage and abuse of patients’ gastrointestinal data. Implementing data encryption and security protocols is a critical step in ensuring data privacy. It ensures data security during data transmission and storage and prevents unauthorized access. Data protection is also a significant concern for AI-based autonomous lesion detection, which heavily relies on extensive training data. The fundamental legislation, the Health Insurance Portability and Accountability Act (HIPAA), has been implemented to protect patient privacy and facilitate efficient data utilization and sharing through de-identification of sensitive information like names and email addresses^174^. However, solely depending on this regulatory framework may not offer comprehensive protection against data breaches, especially considering the potential risk of re-identification through the aggregation of multiple datasets. Federated machine learning is a promising privacy protection strategy. It performs data training iterations locally and only returns computation results to the central database for integration^175^. This distributed system, along with further local algorithm encryption including differential privacy, homomorphic encryption, and collective learning, can significantly reduce the risk of data leakage.

### Interference with human tissue

When carrying out experimental verification of WCE intelligent technologies on animals or human bodies, the final results may deviate significantly from homogeneous models. This originates from the impact of unique physical properties of the biological body^116^, the irregular movement of gastrointestinal tissues^75^, complex fluid environments^106,176^, and other unknown factors. In order to accurately evaluate each interference and optimize theoretical values, it is necessary to conduct a comparative analysis among different models. The required experimental procedure is summarized in Fig. 6d.

### Data diversity

This index mainly targets AI-based autonomous lesion detection technology. It is well acknowledged that the dataset profoundly influences the precision of deep learning algorithms. When working with a small sample size, the incumbent model is prone to overfitting. Mitigating this issue is possible by amplifying the sample size. Nevertheless, data sourced from a singular agency or sampled from a restricted geographical region may inadvertently introduce bias and compromise generalizability^177^. Consequently, to successfully deploy AI in autonomous wound detection in a large-scale clinical setting, it becomes indispensable to amalgamate data from varying regions and ethnicities to enable data sharing.

### Easy manipulation

Operating multi-functional capsule robots within the gastrointestinal tract presents a substantial challenge for clinicians without proper training. Although commercial magnetic field active drive systems enable basic capsule motion control (such as translation and rotation) using two joysticks^37^, they are not suitable for advanced capsules. Therefore, it is crucial to revise the control methods and evaluate the complexity of doctors’ operations before integrating intelligent capsule robots into clinical practice. According to Hager et al.^178^, the ideal control method should permit doctors to manage a slave robotic system intuitively, employing identical actions, gestures, or tools. This process would necessitate a forceful human-robot interactivity involving visual and tactile feedback. Based on the criterion, several suitable control tactics have been explored.

For active magnetic field drive, there are two main methods: notably human-robot collaboration (HRC) and teleoperation (TO). In HRC, the operators manually control the capsule’s movement by manipulating the actuator at the endpoint of the mechanical arm, which is equipped with a force/torque sensor. Due to the direct contact with the patient, they are fully aware of the surrounding environment. TO employs a six-degree-of-freedom tactile device (Phantom Omni) to interact kinematically with an intricate virtual environment, thus offering an authentic 3D navigation experience^179^. Statistical analysis of control parameters confirmed that TO control surpasses HRC control in reliability, rendering it better suited for conducting steady robot-assisted capsule endoscopic surgery^180^. Besides, the Korean Society of Biomedical Engineering has also developed a virtual reality-combined endoscope interface system based on a head-mounted display device^181^. Equipped with a gyroscope sensor, this system manipulates the capsule endoscope’s orientation via an electromagnetic drive system.

For magnetic-controlled diagnosis and treatment, the customized remote operating system (ROS) is more recommendable. ROS comprises a primary system that includes the main capsule robot and a magnetic positioning system, as well as a subordinate system involving an auxiliary robot, magnetic drive, and positioning systems. As the main and auxiliary capsules synchronize their morphology and movement, the manual operation of the primary capsule will guide the subordinate capsule’s movement. This approach has proven feasible in ex-vivo pig stomach experiments^150^.

If assessing which control method is least challenging for doctors, multiple volunteers could undergo training for each technical solution, and the time index would illuminate their differences. In our opinion, the combination of TO and ROS may be the optimal manipulation scheme for a “capsule surgeon”.

## Discussion and outlook

### Comparison with other publications

Recently, there have been some publications introducing the latest advancements in technologies related to WCE (detailed summary seen in Supplementary Table 3). For instance, Steiger et al.^182^ focused on the progress in ingestible electronic devices for diagnostic and therapeutic purposes. Abdigazy et al.^183^ discussed the end-to-end design of seven key components including sensors, actuators, and integrated circuits. Comparatively speaking, our work offers a more critical and comprehensive evaluation of the “capsule surgeon” concept, emphasizing intelligent robotic technologies. We not only assess the strengths and weaknesses of different WCE robot proposals but also highlight the potential of six breakthrough intelligent technologies, particularly AI-based autonomous lesion detection. Furthermore, the translational strategies for clinical integration are also a noteworthy aspect.

### Current challenges and future directions

WCE technology has progressed from its preliminary role as a “gastrointestinal video recorder” to its current state as a “controllable diagnostic capsule robot”. Notably, breakthroughs in multidisciplinary fields such as AI, MEMS, and biomedicine, have enabled the emergence of intelligent capsule robots in recent years. Although most explorations into the intelligence capabilities of WCE devices have been limited to tests with pig models or isolated gastrointestinal tracts, their clinical application is a promising prospect for the future.

To advance this progress, it is crucial to not only focus on the performance optimization of various WCE intelligent technologies but also recognize the challenges arising from multi-disciplinary integration. One key task involves addressing technical compatibility, particularly in minimizing interference between different magnetic field-based technologies. Simultaneous operation of WPT, magnetic field active drive, and magnetic positioning may lead to unexpected deviations in magnetic field control and measurement. Pre-simulating such scenarios and exploring magnetic field decoupling methods are essential. The synchronous magnetic actuation and positioning strategy proposed by Xu et al.^184^ provides a valuable solution. Another consideration is the technological complexity, which often hinders performance. For example, embedding intelligent algorithms into the capsule’s chips is the ultimate goal of AI-based autonomous lesion detection, which could avoid delay mistakes from remote forecasting. However, the extensive parameters in the algorithm make flawless implementation difficult with limited hardware resources. Balancing inference accuracy, memory utilization rate of model parameters, inference speed, and power consumption are the primary focus. Knowledge distillation^185^ and lightweight CNN^186^ are worthy of reference, although they have not fully achieved the objective of local running.

**Fig. 7:**
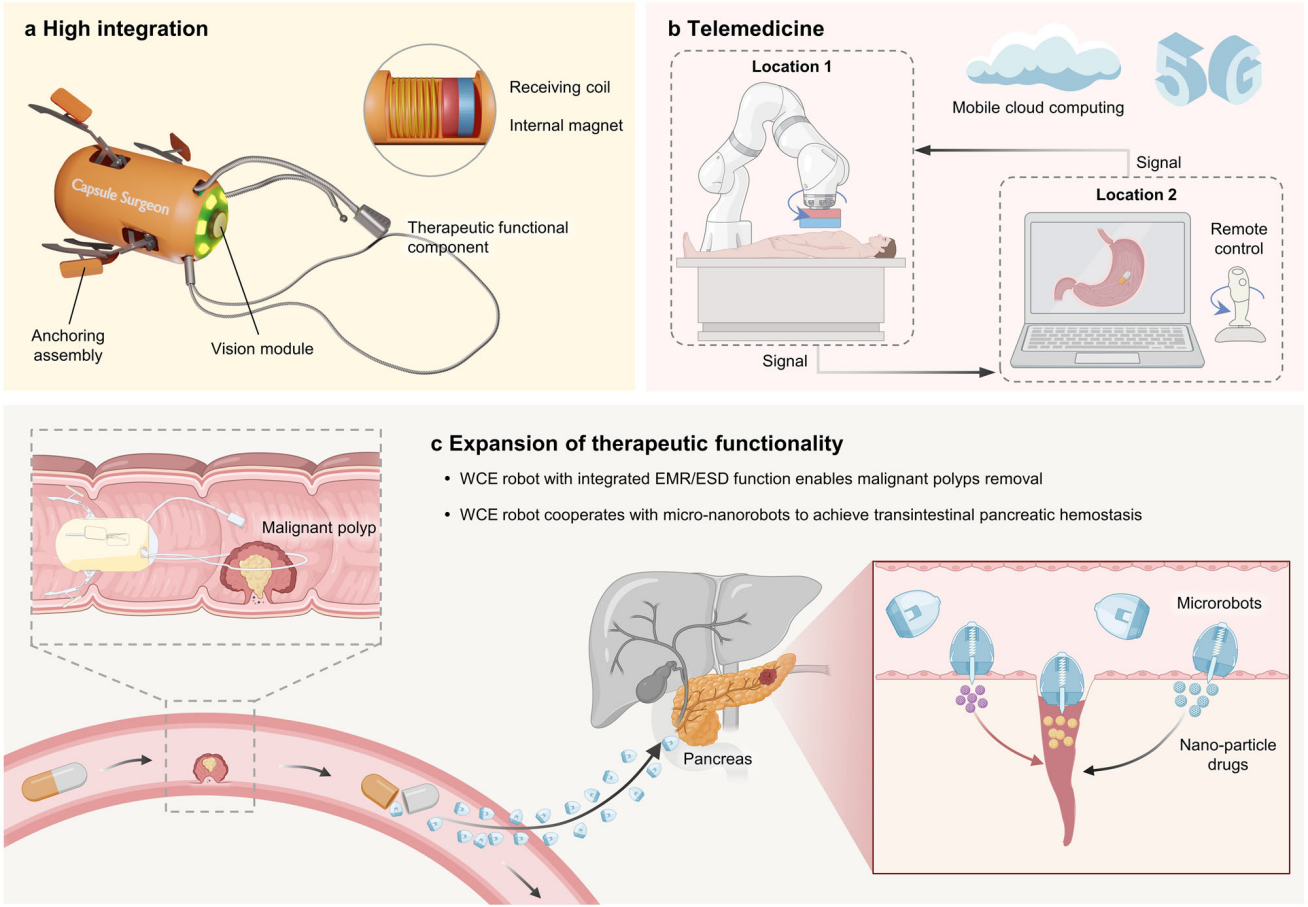
Key technological advancements required for future developments in WCE. **a** Achieving high integration. A plausible approach would be to fit magnets and coils within the capsule for WPT, active motion, positioning, diagnosis, and treatment under an external magnetic field. **b** Telemedicine. The incorporation of mobile cloud computing coupled with 5G communication could facilitate the telemedicine function. **c** Expansion of therapeutic functionality of WCE robots. It is not confined to malignant polyp removal and multi-scale robotic coordination for carrying out treatments in areas that are normally inaccessible. EMR endoscopic mucosal resection, ESD endoscopic submucosal dissection. Figure 7, created with BioRender.com, released under a Creative Commons Attribution-NonCommercial-NoDerivs 4.0 International license.

In addition, as the long-term development goals of WCE robots, the following three vital areas are recommended to be considered (Fig. 7):

1. High integration. Despite its advantages over traditional wired endoscopes, capsule robots’ functional integration remains limited. A typical functional structure occupies a lot of space in the capsule, even making the capsule too large to swallow^187^. While integrating these novel technologies remains a complex issue, a potential solution might employ single-drive technology to perform multiple functions. Among other options, magnetic driving is notable for its ability to eliminate complicated mechanisms, reducing the need for an on-board power source, thus decreasing the device’s overall size and intricacy. Another method to overcome the spatial limitation might involve employing swarm capsule robots, each with a specific function. Although current studies are exploring the cooperative operations of multiple capsule robots^188,189^, the initial outcomes are not entirely promising.
2. Telemedicine: Throughout the COVID-19 pandemic, numerous telemedicine initiatives emerged, equipping clinicians with the ability to treat patients remotely from their own homes^190^. Leveraging recent advancements in mobile cloud assistance and 5G communication, such a model could be efficiently introduced to the field of WCE. This approach not only mitigates risks associated with exposure to high-risk patients but also enables access to clinical diagnostics and treatment by gastroenterology specialists for residents in less developed regions.
3. Expansion of therapeutic functionality: WCE robots are currently used primarily for diagnostic and treatment functions such as biopsy and medication administration, without the capability for total removal of diseased tissue. Endoscopic mucosal resection (EMR) and endoscopic submucosal dissection (ESD) have established themselves as the gold standard for minimally invasive resection of early malignant gastrointestinal tract lesions, commonly integrated into wired endoscopes^191^. Technological advancements, especially in power supply, hold the potential to incorporate EMR and ESD functionalities into capsules, significantly broadening their field of clinical application. Moreover, the effectiveness of medical micro/nanorobots in the areas of treatment, surgery, diagnosis, and medical imaging has been validated^192^. A larger-scale WCE robot can serve as a “carrier battleship”, while clusters of micro/nanorobots act as “soldiers” executing tissue treatment tasks. This strategy not only exploits the benefits of multi-scaling but also opens possibilities for the exploration of locations within the digestive system (like the pancreas) that WCE robots could not previously access.

Achieving the vision mentioned necessitates considerable technical iteration. Recent advancements in design patterns for integrating WCE with additional functionalities can offer valuable insights, even if they do not focus on equipping a camera or maintaining the capsule shape. For instance, ingestible electroceutical capsules with circumferential electrodes on the shell surface^193^ or conductive hooked probes integrated within^194^ help regulate hormone release and enhance gastrointestinal tissue activity. This electrostimulation therapy presents a novel research avenue for WCE robots in treating chronic conditions such as diabetes, obesity, and mild gastroparesis. Subsequently, it is necessary to add magnetically soft materials within the capsules to replace conventional hard magnets. Recent studies have explored using a double magnetic film as a soft valve for dual drug release^195^ and multi-layered magnetically soft robots for adhering to internal wounds at various target points^196^. Magnetic films not only offer integration benefits but also expand the therapeutic capabilities of WCE through their multimodal response to magnetic fields. Moreover, inflatable capsule robots that use magnetic fields to trigger chemical reactions have been shown to be viable for weight loss^197^ and intestinal hemostasis^198^.

In this transformative era, it could be predicted that the next decade of WCE technology will see the maturation of capsule robots into “capsule surgeons”. Once ingested by the patient, these robots would have the capability to self-navigate and execute minimally invasive treatment within the gastrointestinal tract, thereby eliminating the necessity for direct physician intervention.

### Reporting summary

Further information on research design is available in the Nature Portfolio Reporting Summary linked to this article.

## Supplementary information

Supplementary Information

Reporting Summary
