# Supplementary material for: Robotic wireless capsule endoscopy: recent advances and upcoming technologies
Source: Nat Commun. 2024 May 30;15:4597. doi: 10.1038/s41467-024-49019-0 (PMC11139981; doi:10.1038/s41467-024-49019-0)
Supplement: Supplementary file 1 — Supplementary Information [file 41467_2024_49019_MOESM1_ESM.pdf]

Supplementary Information

Robotic wireless capsule endoscopy: recent advances and upcoming technologies

Qing Cao[1,2], Runyi Deng[1,2], Yue Pan[1,2], Ruijie Liu[1,2], Yicheng Chen[3], Guofang Gong[1,2], Jun Zou[1,2], Huayong Yang[1,2], Dong Han[1,2*]

[1]State Key Laboratory of Fluid Power and Mechatronic Systems, Zhejiang University, Hangzhou, 310027, China

[2]School of Mechanical Engineering, Zhejiang University, Hangzhou, 310027, China

[3]Sir Run-Run Shaw Hospital, College of Medicine, Zhejiang University, Hangzhou, 310016, China

[*]Corresponding author: dong_han@zju.edu.cn

Contents

Supplementary Table 1: Commercially WCE devices **3**

Supplementary Table 2: AI-based WCE pathology recognition models (2018~2024) **4**

Supplementary Table 3: Summary of review papers on WCE and ingestible electronics (2018~2024) **5**

References **6-8**

Supplementary Table 1. Commercially WCE devices.

Device		Dimensions (mm)	Field of view (°)	Resolution (pixels)	Image transfer rate (fps)	Battery life (h)	Active locomotion	Communication mode	Location mode	Intelligent assistive reading tool
PillCam™ (Medtronic)	SB3	11×26	156	320×320	2 or 2~6	≥8	×	RF	×	QuickView
	Colon2	11×26	172	N/A	4~35	≥10	×	RF	×	QuickView
	Crohn	12×32	168	N/A	4~35	≥10	×	RF	×	QuickView
MiroCam® (Intromedic)	Navi	11×24	170	320×320	3	9	Magnetic (handheld)	IBC	×	Express View
	MC1600	11×24	170	320×320	6	12	Magnetic (handheld)	IBC	×	Express View
	MC2000	11×30	340	320×320	6	12	×	IBC	×	Express View
OLYMPUS EC-S10 (Olympus)		11×26	160	1920×1080	2	12	×	RF	3D Track	Omni
OMOM® (JINSHAN)	SC100	13×28	172	512×512	2~10	12	×	RF	×	SSAR
	RC100	12×30	160	512×512	2 or 2~8	11	Magnetic (robotic)	RF	MSL	SSAR
	CC100	12×32	344	360×360	4~35	10	×	RF	×	SSAR
CapsoCam® Plus (CapsoVision)		11×31	360	221×184	3 or 5	15	×	USB	×	CapsoView®
NaviCam® (Ankon)	SB	12×27	160	640×480	0.5~15	>16	×	RF	TVS	ProScan™
	Stomach	12×27	160	640×480	0.5~6	>16	Magnetic (robotic)	RF	MSL	ESview™
	Colon	12×31	320	640×480	0.5~17, 2~38	10	×	RF	TVS	×

Blue means the element is not applicable, red means the element is applicable.

Abbreviations: N/A, information not available; RF, radio frequency; IBC, intrabody communication; 3D, three-dimensional; SSAR, SmartScan-assisted reading; MSL, magnetic scan locator; TVS, topographical video segmentation; USB, universal serial bus; JINSHAN, JinShan Science & Technology; Ankon, Ankon Technologies.

Supplementary Table 2. AI-based WCE pathology recognition models (2018~2024).

References	Data sources	AI algorithms	Applications		Evaluation metrics			
					Accuracy	Sensitivity (Recall)	Specificity	Others
Amiri et al.[1]	KID[2]	SVM	Bleeding	Detection and classification	0.982	0.988	N/A	Precision: 0.975, F1-score: 0.982
Pannu et al.[3]	KID[2]	CNN			0.95	0.92	0.96	Precision: 0.91, F1-score: 0.923
Patel et al.[4]	Private: University Hospital of Coimbra, Portugal	SVM			0.9818	0.98	N/A	Precision: 0.98, F1-score: 0.98
Rustam et al.[5]	Private: Sheikh Zayed Hospital Rahim Yar Khan, Pakistan	MobileNet, CNN			0.993	0.994	N/A	Precision: 1, F1-score: 0.997, Cohen's kappa: 0.995
	Google search engine				0.978	0.96	N/A	Precision: 0.99, F1-score: 0.979, Cohen's kappa: 0.957
Garbaz et al.[6]	KID[2]	Inception-ResNet-V2, CNN			0.985	0.985	0.99	Precision: 0.985
Ghosh et al.[7]	https://www.capsuleendoscopy.org, KID[2]	AlexNet			0.9944	0.9751	0.9988	Precision: 0.995, F1-score: 0.9849
		SegNet		Segmentation	0.9442	N/A	N/A	Weight IoU: 0.9069
Kanakatte et al.[8]	KID[2]	Compact U-Net			0.959	0.9957	0.91	N/A
	https://sites.google.com/site/farahdeeba073/Research/resources				N/A	N/A	N/A	Dice-score: 0.91
Hajabdollahi at al.[9]	KID[2], Bleeding images[10]	MLP			N/A	N/A	N/A	Dice-score:0.831, AUC: 0.974
		CNN			N/A	N/A	N/A	Dice-score:0.869, AUC: 0.985
Garbay et al.[11]	Private: Hospital Clinic, Barcelona, Spain	VGG-16	Polyps	Detection and classification	0.9894	0.9905	0.9834	N/A
		Squeezenet			0.9794	0.9873	0.9382	N/A
Yang et al.[12]	Private: Hospital, KVASIR[13]	SVM			0.96	0.958	0.962	Processing time: 38 min
Souaidi et al.[14]	http://www.endoatlas.org/	Inception V3, SVM			0.989	N/A	N/A	Precision: 0.98, F1-score: 0.98
Ito et al.[15]	KVASIR[13]	YOLO V5			0.77	1	N/A	Precision: 0.68, F1-score: 0.81
Souaidi et al.[16]	WCE images[17], ETIS-larib (https://polyp.grand-challenge.org/EtisLarib/), CVC-ClinicDB (https://polyp.grand-challenge.org/CVClinicDB)	Inception V4			N/A	N/A	N/A	Precision: 0.9329, Speed: 44.5 FPS
Belabbes et al.[18]	WCE images[17]	VGG-16			N/A	N/A	N/A	Precision: 0.9432, F1-score:0.9137
	CVC-ClinicDB				0.9092	0.8895	N/A	F1-score:0.8962
	KVASIR[13]				0.9019	0.9156	N/A	F1-score:0.9196
	ETIS-larib				0.9054	0.8882	N/A	F1-score:0.8949
Nadimi[19]	Private: Hospital	ZF-Net			0.946	0.953	0.928	N/A
Sornapudi et al.[20]	MICCAI[21], GIANA (https://giana.grand-challenge.org), CVC-ClinicDB	Region-based CNN		Segmentation	N/A	0.9552	N/A	Precision: 0.9846, F1-score: 0.9667
Afonso et al.[22]	Private: São João University Hospital, Porto, Portugal	CNN	Ulcer	Detection and classification	0.956	0.908	0.971	N/A
Masmoudi et al.[23]	KVASIR[13]	ResNet50, ResNet152-V2, GA			0.9967	N/A	N/A	N/A
Alaskar[24]	http://www.drkhuroo.in/#	AlexNet, GoogleNet			1	1	1	N/A
Khan et al.[25]	Private: POF Hospital, Pakistan	Mask-RCNN		Segmentation	0.8808	N/A	N/A	N/A
		MSVM	Multiple types of lesions	Detection and Classification	0.9992	N/A	N/A	N/A
Valério et al.[26]	GIANA	DenseNet-161			N/A	0.93	N/A	Precision: 0.94, AUC: 0.87
Kundu et al.[27]	http://www.capsuleendoscopy.org	SVM			0.84~0.9837	0.7~1	0.8571~1	-
Guo et al.[28]	CAD-CAP (endoscopy centers in France)	EfficientNet			0.9611	0.9833	N/A	F1-score: 0.9916
Su et al.[29]	KVASIR[13]	DCNN			0.948	N/A	N/A	-
Sharma et al.[30]	KVASIR[13]	VGG-16, InceptionV3, ResNet50			0.9908	0.99	N/A	Precision: 1
Oh et al.[31]	Private: Dongguk University Ilsan Hospital	Transformer			N/A	0.951	0.834	-
Vieira et al.[32]	KID[2]	Mask-RCNN, PANet		Segmentation	0.6995	0.5584~0.8452	N/A	Precision: 0.7333~1, F1-score: 0.6667~0.9045
Amiri et al.[33]	GIANA, KID[2], KVASIR[13]	SVM			0.913	0.89	N/A	F1-score: 0.885, Dice-score: 0.8703, IoU: 0.7692
Jeon et al.[34]	Private: WCE images from 52 patients	GoogLeNet, SVM	Lesions	Detection and Classification	0.9856	0.997	0.9743	N/A
Gao et al.[35]	Private: BeijingJiShuiTan Hospital, China	CNN, LSTM			0.9327	0.8617	0.9541	N/A
Mascarenhas et al.[36]	Private: São João University Hospital (Porto, Portugal), Private: ManopH Gastroenterology Clinic (Porto, Portugal)	CNN	Colonic lesions	Detection and Classification	0.953	0.9	0.991	AUC: 0.99
Sundaram et al.[37]	-	K-means, SVM2	Tumors	Detection and classification	0.957	0.96	0.954	N/A
Gan et al.[38]	Private: The West China Hospital, China	CNN	Hookworms	Detection and Classification	0.912	0.922	0.911	AUC: 0.972
Koh et al.[39]	Private: Hospital	DT, SVM, KNN, Adaboost, Bagged trees, Discriminant subspace	Celiac disease	Detection and classification	0.8889	0.8967	0.8667	Lesions multiclass classification accuracy: 0.72
Marin-Santos et al.[40]	Private: Juan Ramón Jiménez hospital in Huelva (Andalusia, Spain)	CNN	Crohn disease	Detection and Classification	N/A	0.95~0.99	0.96~0.99	AUC: 0.9973

Blue represents deep learning algorithms, red represents traditional machine learning algorithms, and green represents mixed algorithms.

Abbreviations: SVM, support vector machine; CNN, convolutional neural network; MLP, multilayer perceptron; MSVM, multi-class SVM; DCNN, deep CNN; LSTM, long short term memory; DT, decision tree; KNN, k-nearest neighbour.

Supplementary Table 3. Summary of review papers on WCE and ingestible electronics (2018~2024)

References	Research fields	Endurance	Active locomotion	Communi cation	Location	AI-based autonomous lesion detection	Diagnostic and therapeutic functions	Translational strategies for clinical integration	Concerns
Steiger et al.[41], 2019	Ingestible electronics	✓	✓	✓	×	×	✓	×	• Diagnostics and therapy
Park et al.[42], 2019	WCE	×	×	×	×	✓	×	×	• AI
Beardslee et al.[43], 2020	Ingestible electronics	×	×	×	×	×	✓	×	• Minimally invasive diagnosis and monitoring
Muhammad et al.[44], 2020	WCE	×	×	×	×	✓	×	×	• Vision-based smart healthcare
Alsunaydih et al.[45], 2021	WCE	×	✓	×	×	×	✓	×	• Sensing, locomotion and navigation
Gayen et al.[46], 2021	WCE	×	×	✓	×	×	×	×	• Miniaturized antennas
Cummins[47], 2021	WCE	×	×	×	×	×	✓	×	• Diagnostics and therapy
Kim et al.[48], 2021	WCE	×	×	×	×	✓	×	×	• AI
Moen et al.[49], 2022	Colonic WCE	×	×	×	×	✓	×	×	• AI
Hanscom et al.[50], 2022	WCE	×	✓	×	✓	✓	✓	×	• Endoscopic capsule robot-based diagnosis, navigation and localization
Zeising et al.[51], 2022	WCE	×	×	×	✓	×	×	×	• Localization of passively WCE
Chen et al.[52], 2022	WCE	✓	✓	×	✓	×	✓	×	• Magnetically actuated capsule robots
Thwaites et al.[53], 2024	Ingestible electronics	×	×	×	×	×	✓	×	• Ingestible sensors, interventional funtions and safety assessment
Abdigazy et al.[54], 2024	Ingestible electronics	✓	✓	✓	✓	×	✓	×	• End-to-end design of ingestible electronics
**Our work, 2024**	**WCE**	✓	✓	✓	✓	✓	✓	✓	• **For intelligent robotic technologies**<br>• **A critical and comprehensive evaluation of the "capsule surgeon" concept**<br>• **Translational strategies for clinical integration**

Blue means the element is not applicable, red means the element is applicable.

References

1. Amiri, Z., Hassanpour, H. & Beghdadi, A. A Computer- Aided Method to Detect Bleeding Frames in Capsule Endoscopy Images. In *2019 8th European Workshop on Visual Information Processing (EUVIP)* 217-221 (IEEE, 2019).
2. Koulaouzidis, A. et al. KID Project: an internet-based digital video atlas of capsule endoscopy for research purposes. *Endosc Int Open* **05**, E477-E483 (2017).
3. Pannu, H.S., Ahuja, S., Dang, N., Soni, S. & Malhi, A.K. Deep learning based image classification for intestinal hemorrhage. *Multimed. Tools Appl.* **79**, 21941-21966 (2020).
4. Patel, A., Rani, K., Kumar, S., Figueiredo, I.N. & Figueiredo, P.N. Automated bleeding detection in wireless capsule endoscopy images based on sparse coding. *Multimed. Tools Appl.* **80**, 30353-30366 (2021).
5. Rustam, F. et al. Wireless Capsule Endoscopy Bleeding Images Classification Using CNN Based Model. *IEEE Access* **9**, 33675-33688 (2021).
6. Garbaz, A., Lafraxo, S., Charfi, S., Ansari, M.E. & Koutti, L. Bleeding classification in Wireless Capsule Endoscopy Images based on Inception-ResNet-V2 and CNNs. In *2022 IEEE Conference on Computational Intelligence in Bioinformatics and Computational Biology (CIBCB)* 1-6 (IEEE, 2022).
7. Ghosh, T. & Chakareski, J. Deep Transfer Learning for Automated Intestinal Bleeding Detection in Capsule Endoscopy Imaging. *J. Digit. Imaging* **34**, 404-417 (2021).
8. Kanakatte, A. & Ghose, A. Precise Bleeding and Red lesions localization from Capsule Endoscopy using Compact U-Net. In *2021 43rd Annual International Conference of the IEEE Engineering in Medicine & Biology Society (EMBC)* 3089-3092 (IEEE, 2021).
9. Hajabdollahi, M. et al. Segmentation of bleeding regions in wireless capsule endoscopy for detection of informative frames. *Biomed. Signal Process. Control* **53**, 101565 (2019).
10. Deeba, F. Bleeding images and corresponding ground truth of CE images. https://sites.google.com/site/farahdeeba073/Research/resources (2016).
11. Garbay, T. et al. Distilling the knowledge in CNN for WCE screening tool. In *2019 Conference on Design and Architectures for Signal and Image Processing (DASIP)* 19-22 (IEEE, 2019).
12. Yang, J., Chang, L., Li, S., He, X. & Zhu, T. WCE polyp detection based on novel feature descriptor with normalized variance locality-constrained linear coding. *Int. J. Comput. Assist. Radiol. Surg.* **15**, 1291-1302 (2020).
13. Pogorelov, K. et al. KVASIR: A Multi-Class Image Dataset for Computer Aided Gastrointestinal Disease Detection. In *Proc. the 8th ACM on Multimedia Systems Conference* 164–169 (ACM, 2017).
14. Souaidi, M. & El Ansari, M. Automated Detection of Wireless Capsule Endoscopy Polyp Abnormalities with Deep Transfer Learning and Support Vector Machines. In *Advanced Intelligent Systems for Sustainable Development* 870-880 (Cham: Springer International Publishing, 2022).
15. Ito, T. et al. Extraction of Non-Diagnosable Images Captured by a Capsule Endoscope and Polyp Detection Using YOLOv5. In *2022 IEEE/SICE International Symposium on System Integration (SII)* 742-747 (IEEE, 2022).
16. Souaidi, M. & El Ansari, M. Multi-Scale Hybrid Network for Polyp Detection in Wireless Capsule Endoscopy and Colonoscopy Images. *Diagnostics* **12**, 2030 (2022).
17. Prasath, V.B.S. Polyp Detection and Segmentation from Video Capsule Endoscopy: A Review. *J. Imaging* **3**, 1 (2017).
18. Belabbes, M.A., Oukdach, Y., Souaidi, M., Koutti, L. & Charfi, S. Advancements in Polyp Detection: A Developed Single Shot Multibox Detector Approach. *IEEE Access* **12**, 19199-19215 (2024).
19. Nadimi, E.S. et al. Application of deep learning for autonomous detection and localization of colorectal polyps in wireless colon capsule endoscopy. *Comput. Electr. Eng.* **81**, 106531 (2020).
20. Sornapudi, S., Meng, F. & Yi, S. Region-Based Automated Localization of Colonoscopy and Wireless Capsule Endoscopy Polyps. *Appl. Sci.* **9**, 2404 (2019).
21. Bernal, J. et al. Comparative Validation of Polyp Detection Methods in Video Colonoscopy: Results From the MICCAI 2015 Endoscopic Vision Challenge. *IEEE Trans. Med. Imaging* **36**, 1231-1249 (2017).

22. Afonso, J. et al. Automated detection of ulcers and erosions in capsule endoscopy images using a convolutional neural network. *Med. Biol. Eng. Comput.* **60**, 719-725 (2022).
23. Masmoudi, Y., Ramzan, M., Khan, S.A. & Habib, M. Optimal feature extraction and ulcer classification from WCE image data using deep learning. *Soft Comput.* **26**, 7979-7992 (2022).
24. Alaskar, H., Hussain, A., Al-Aseem, N., Liatsis, P. & Al-Jumeily, D. Application of Convolutional Neural Networks for Automated Ulcer Detection in Wireless Capsule Endoscopy Images. *Sensors* **19**, 1265 (2019).
25. Khan, M.A. et al. Gastrointestinal diseases segmentation and classification based on duo-deep architectures. *Pattern Recognit. Lett.* **131**, 193-204 (2020).
26. Valério, M.T., Gomes, S., Salgado, M., Oliveira, H.P. & Cunha, A. Lesions Multiclass Classification in Endoscopic Capsule Frames. *Procedia Comput. Sci.* **164**, 637-645 (2019).
27. Kundu, A.K., Fattah, S.A. & Wahid, K.A. Multiple Linear Discriminant Models for Extracting Salient Characteristic Patterns in Capsule Endoscopy Images for Multi-Disease Detection. *IEEE J. Transl. Eng. Health Med.-JTEHM* **8**, 1-11 (2020).
28. Guo, X. et al. Multiple abnormality classification in wireless capsule endoscopy images based on EfficientNet using attention mechanism. *Rev. Sci. Instrum.* **92**, 094102 (2021).
29. Su, Q. et al. Deep convolutional neural networks with ensemble learning and transfer learning for automated detection of gastrointestinal diseases. *Comput. Biol. Med.* **150**, 106054 (2022).
30. Sharma, A., Kumar, R. & Garg, P. Deep learning-based prediction model for diagnosing gastrointestinal diseases using endoscopy images. *Int. J. Med. Inf.* **177**, 105142 (2023).
31. Oh, S. et al. Video Analysis of Small Bowel Capsule Endoscopy Using a Transformer Network. *Diagnostics* **13**, 3133 (2023).
32. Vieira, P.M. et al. Multi-pathology detection and lesion localization in WCE videos by using the instance segmentation approach. *Artif. Intell. Med.* **119**, 102141 (2021).
33. Amiri, Z., Hassanpour, H. & Beghdadi, A. Abnormalities detection in wireless capsule endoscopy images using EM algorithm. *Visual Comput.* **39**, 2999-3010 (2023).
34. Yejin, J. et al. Deep convolutional neural network-based automated lesion detection in wireless capsule endoscopy. In *International Forum on Medical Imaging in Asia 2019* 292-296 (SPIE, 2019).
35. Gao, Y., Lu, W., Si, X. & Lan, Y. Deep Model-Based Semi-Supervised Learning Way for Outlier Detection in Wireless Capsule Endoscopy Images. *IEEE Access* **8**, 81621-81632 (2020).
36. Mascarenhas, M. et al. Performance of a Deep Learning System for Automatic Diagnosis of Protruding Lesions in Colon Capsule Endoscopy. *Diagnostics* **12**, 1445 (2022).
37. Shanmuga Sundaram, P. & Santhiyakumari, N. An Enhancement of Computer Aided Approach for Colon Cancer Detection in WCE Images Using ROI Based Color Histogram and SVM2. *J. Med. Syst.* **43**, 29 (2019).
38. Gan, T. et al. Automatic Detection of Small Intestinal Hookworms in Capsule Endoscopy Images Based on a Convolutional Neural Network. *Gastroenterol. Res. Pract.* **2021**, 5682288 (2021).
39. Koh, J.E.W. et al. Automated interpretation of biopsy images for the detection of celiac disease using a machine learning approach. *Comput. Methods Programs Biomed.* **203**, 106010 (2021).
40. Marin-Santos, D., Contreras-Fernandez, J.A., Perez-Borrero, I., Pallares-Manrique, H. & Gegundez-Arias, M.E. Automatic detection of crohn disease in wireless capsule endoscopic images using a deep convolutional neural network. *Appl. Intell.* **53**, 12632-12646 (2023).
41. Steiger, C. et al. Ingestible electronics for diagnostics and therapy. *Nat. Rev. Mater.* **4**, 83-98 (2019).
42. Park, J. et al. Recent Development of Computer Vision Technology to Improve Capsule Endoscopy. *Clin. Endosc.* **52**, 328-333 (2019).
43. Beardslee, L.A. et al. Ingestible Sensors and Sensing Systems for Minimally Invasive Diagnosis and Monitoring: The Next Frontier in Minimally Invasive Screening. *ACS Sens.* **5**, 891-910 (2020).
44. Muhammad, K., Khan, S., Kumar, N., Del Ser, J. & Mirjalili, S. Vision-based personalized Wireless Capsule

Endoscopy for smart healthcare: Taxonomy, literature review, opportunities and challenges. *Futur. Gener. Comp. Syst.* **113**, 266-280 (2020).
45. Alsunaydih, F.N. & Yuce, M.R. Next-generation ingestible devices: sensing, locomotion and navigation. *Physiol. Meas.* **42**, 04TR01 (2021).
46. Gayen, S., Biswas, B. & Karmakar, A. The quest for a miniaturized antenna in the wireless capsule endoscopy application: a review. *Int. J. Microw. Wirel. Technol.* **14**, 1195-1205 (2022).
47. Cummins, G. Smart pills for gastrointestinal diagnostics and therapy. *Adv. Drug Deliv. Rev.* **177**, 113931 (2021).
48. Kim, S.H. & Lim, Y.J. Artificial Intelligence in Capsule Endoscopy: A Practical Guide to Its Past and Future Challenges. *Diagnostics* **11**. 1722 (2021).
49. Moen, S., Vuik, F.E.R., Kuipers, E.J. & Spaander, M.C.W. Artificial Intelligence in Colon Capsule Endoscopy—A Systematic Review. *Diagnostics* **12**, 1994 (2022).
50. Hanscom, M. & Cave, D.R. Endoscopic capsule robot-based diagnosis, navigation and localization in the gastrointestinal tract. *Front. Robot. AI* **9**, 896028 (2022).
51. Zeising, S., Thalmayer, A.S., Lübke, M., Fischer, G. & Kirchner, J. Localization of Passively Guided Capsule Endoscopes—A Review. *IEEE Sens. J.* **22**, 20138-20155 (2022).
52. Chen, W., Sui, J. & Wang, C. Magnetically Actuated Capsule Robots: A Review. *IEEE Access* **10**, 88398-88420 (2022).
53. Thwaites, P.A. et al. Review article: Current status and future directions of ingestible electronic devices in gastroenterology. *Aliment. Pharmacol. Ther.* **59**, 459-474 (2024).
54. Abdigazy, A. et al. End-to-end design of ingestible electronics. *Nat. Electron.* **7**, 102-118 (2024).
